# The role of the electroencephalogram (EEG) in determining the aetiology of psychosis: a systematic review and meta-analysis of diagnostic test accuracy☆

**DOI:** 10.1016/j.schres.2025.07.020

**Published:** 2025-07-31

**Authors:** Jack B. Fanshawe, Rosie Lawrence, Dory A. Ghanem, Lucy Pauli, Rebecca Whincup, Emma Salter, Brendan F. Sargent, Lauren Evans, Iman Jasani, Dominic Oliver, Joseph Hutchinson, Steven White, Ben Carter, Anthony S. David, Glyn Lewis, Michael S. Zandi, Jonathan P. Rogers

**Affiliations:** a Department of Psychiatry, University of Oxford, Oxford, UK; b Oxford Health NHS Foundation Trust, Oxford, UK; c Division of Psychiatry, University College London, London, UK; d Medical School, University College London, London, UK; e Sheffield Health and Social Care NHS Foundation Trust, UK; f Leicestershire Partnership NHS Trust, Leicester, UK; g South London and Maudsley NHS Trust, London, UK; h Department of Clinical Neurophysiology, The National Hospital for Neurology and Neurosurgery, London, UK; i Department of Biostatistics and Health Informatics, King’s College London, London, UK; j National Hospital for Neurology and Neurosurgery, London, UK; k Queen Square Institute of Neurology, University College London, London, UK; l Department of Neuropsychiatry, National Hospital for Neurology and Neurosurgery, London, UK

**Keywords:** EEG, Psychosis, Systematic review, Meta-analysis, Schizophrenia

## Abstract

**Background:**

Psychosis is a transdiagnostic condition which can manifest as part of many disorders including those of primary psychiatric origin, or secondary to a medical condition. This study aims to systematically review and quantify the diagnostic accuracy of electroencephalography (EEG) in identifying people with psychosis secondary to a medical condition.

**Methods:**

A comprehensive search strategy was implemented across multiple databases. Inclusion criteria specified observational human studies with a minimum of 20 participants experiencing psychosis who underwent EEG. The reference standard was the final clinical diagnosis. Two reviewers independently assessed article inclusion using predefined eligibility criteria. Data were extracted by two blinded reviewers. A bivariate meta-analysis was conducted to model the correlation between sensitivity and specificity.

**Findings:**

The search strategy yielded 8688 results, of which 38 were eligible. The main diagnostic test accuracy meta-analysis found that the sensitivity (i.e., the proportion of patients with secondary psychosis who had an abnormal EEG) was 0·71 (95 % CI 0·61–0·80) and the specificity (i.e., the proportion of patients with primary psychosis who had a normal EEG) was 0·67 (95 % CI 0·58–0·74). The area under the receiver operating characteristics curve was 0·67, corresponding to poor discrimination. The pattern for each of the specific EEG abnormalities was a low-moderate sensitivity and high specificity.

**Interpretation:**

EEG demonstrates poor discrimination in identifying a secondary psychosis. Specific EEG abnormalities exhibit potential diagnostic value, encouraging more detailed reporting standards. While EEG alone does not definitively distinguish psychosis aetiology, its integration with clinical assessment may enhance diagnostic precision.

## 1 Introduction

Psychosis is a transdiagnostic condition characterised by delusions, hallucinations and disorganised thoughts, with an estimated lifetime prevalence of 7·49 per 1000 people (interdecile range 3.62 to 19.67 per 1000) (Moreno-Küstner et al., 2018). Psychosis most commonly manifests as part of a primary psychiatric disorder, such as schizophrenia or bipolar affective disorder (Maj et al., 2021). However, a significant minority of cases may be due to an underlying medical condition or substance misuse and can be described as, secondary psychosis (Keshavan and Kaneko, 2013). Meta-analytic estimates suggest a secondary cause underpins psychosis in 11 % (95 % CI: 7–16) of cases (Blackman et al., 2024). On presentation, and during the early course of the illness, it can be challenging for clinicians to discern what is the underlying cause of psychosis.

Electroencephalography (EEG) is a neurophysiological tool which records electrical activity generated by brain structures. EEG benefits from being a non-invasive, inexpensive tool which, in comparison to neuroimaging techniques using ionising radiation, poses little risk to those being investigated. Despite this, its use in clinical psychiatry is infrequent and inconsistent (Boutros et al., 2011). Mismatch negativity evidenced by EEG can effectively differentiate patients with schizophrenia from healthy controls (Kim et al., 2019a). Furthermore, research has shown that in psychotic disorders, clinical EEG abnormalities may predict transition to psychosis in people with an at-risk mental state (Gschwandtner et al., 2009), worse outcomes in patients with first-episode psychosis (Manchanda et al., 2008), and treatment response in schizophrenia (Hasey and Kiang, 2013). A recent meta-analysis also explored the value of EEG in establishing the disorder underlying catatonia (Hosseini et al., 2023), finding that an abnormal EEG predicted a secondary (general medical) cause of catatonia with a sensitivity of 0·82 and a specificity of 0·66. It also reported that certain EEG abnormalities (features suggestive of limbic encephalitis, epilepti-form discharges, focal abnormality, or status epilepticus) were highly specific to secondary catatonia.

However, research to date is unclear on whether EEG is useful in ascertaining the aetiology of psychosis. Aetiology can have profound implications for the course and treatment of an individual experiencing psychosis, and subsequently their recovery and outcome. The British Association for Psychopharmacology (BAP) guidelines for schizophrenia recommend that clinicians consider the use of EEG at the stage of assessment and diagnosis (Barnes et al., 2020); the World Federation of Societies for Biological Psychiatry recommends the use of EEG when the clinical picture is unclear, or when there are abnormal findings from a routine examination (Falkai et al., 2005); and the American Psychiatric Association supports the use of EEG if indicated on the basis of neurological examination or history (Keepers et al., 2020). However, there is little evidence on which to base these recommendations.

There is some controversy surrounding the functional-organic distinction and the terminology used around it, which has been criticized for oversimplifying complex and nuanced disease states (Bell et al., 2020). In the current paper, the authors have opted to use the term ‘primary’ to refer to psychoses experienced as part of a mental health condition, and ‘secondary’ for those with an identifiable neuropathological process, underlying general medical or ‘organic’ cause. While acknowledging that the terminology is imperfect, we have adopted terms used in the DSM-5-TR for the sake of clarity and consistency with the rest of the literature (American Psychiatric Association, 2022).

In the current paper, we conducted a systematic review and meta-analysis on the diagnostic test accuracy of EEG in determining the aetiology of psychosis. We aimed to investigate if EEG is a useful tool for clinicians determining if psychosis is of a primary or secondary origin.

## 2 Methods

This systematic review is reported according to PRISMA guidelines (see Supplementary Table S1) (Rethlefsen et al., 2021), and the study protocol was preregistered on PROSPERO (CRD42023398676).

### 2.1 Search strategy

In this systematic review and meta-analysis of diagnostic test accuracy, the authors used MEDLINE, PsycINFO and AMED. The search was run on the 07/12/2023. The full search strategy is presented in the supplementary materials and the search strategy for the Medline database is presented in the panel as follows:

In addition to searching databases, the authors examined reference lists of included articles and contacted significant researchers in the field to identify further works. Duplicate articles were identified automatically using Rayyan software (Ouzzani et al., 2016), followed by manual deduplication comparing similar article citations.

### 2.2 Selection criteria

Eligibility criteria for inclusion of studies were developed using the Cochrane Handbook for Systematic Reviews of Diagnostic Test Accuracy (Leeflang et al., 2023). Included studies were observational human studies published in a peer-reviewed journal after 1980 in English, French, German or Japanese.

Included participants were patients who had a psychotic disorder. In studies where psychiatric presentations were presented at the group level given without a breakdown of EEG findings by diagnosis, we specified that included studies must have a minimum of 90 % of subjects with a psychotic disorder, as a means of balancing the applicability of the study to the relevant population against the risk of bias in losing data (McKenzie et al., 2023). The minimum number of individuals with psychosis who had an EEG was set at 20 to minimise reporting bias.

Included studies required an index test of a clinical scalp EEG result, which was reported at a minimum as normal or abnormal. We excluded EEGs that were reported only during the ictal phase of electroconvulsive therapy (ECT) or other induced seizures, EEGs described only in terms of the absence of a particular abnormality (e.g., no epileptiform activity) and sleep EEGs. Review articles, conference abstracts, and case reports were excluded.

Inclusion criteria for our target condition were individuals diagnosed with a secondary psychotic disorder, which included drug-induced psychosis, and/or a primary (psychiatric) psychotic disorder. Our reference standard was diagnosis in the considered clinical opinion of the report authors, as reported in the paper, at least stating whether the diagnosis was primary psychiatric or secondary to a general medical condition.

Two reviewers (I.J., B.F.S., D.A.G., E.S., J.B.F., J.P.R., L.P., R.L., I.J., and R.W.) independently and with blinding evaluated the eligibility of each article by sequentially reviewing titles and abstracts in parallel; where there was disagreement over whether to include an abstract, a third reviewer (L.E.) arbitrated. Full texts were also reviewed by two authors in a blinded process, supported by arbitration by a third individual in cases of disagreement.

### 2.3 Data extraction

Definitions of each variable for which data were extracted are included in Supplementary Table 2. Data were extracted by two blinded reviewers (B.S., D.A.G., E.S., J.B.F., J.P.R., L.P., R.L., and R.W.) in parallel onto a pre-piloted spreadsheet. Where there were discrepancies between the data extracted, a third author from this list arbitrated.

To consistently classify EEG findings, two clinical neurophysiologists (J.H. and S.W.) developed a template similar to that used in a previous meta-analysis of EEG findings in a psychiatric disorder (Keepers et al., 2020), which was adapted for psychosis (Supplementary Table 4). All EEG reports were coded using this template by a psychiatrist (J.P.R.) and a clinical neurophysiologist (J.H.); an experienced clinical neurophysiologist arbitrated (S.W.). Original authors were contacted for additional data where required. Additional data were provided by Restrepo-Martinez et al. (2020) (Restrepo-Martinez et al., 2020).

Two trained reviewers (B.S., D.A.G., E.S., L.P., and R.L.) assessed each paper using the QUADAS-2 tool for systematic reviews of diagnostic accuracy studies to assess risk of bias and applicability (Whiting et al., 2011; Page et al., 2017). In the case of disagreement, decisions were reconciled with a third reviewer. The tool was adapted to include review-specific guidance (Supplementary Table 5), as recommended within the QUADAS-2 guidance.

### 2.4 Analysis

Where studies were clinically homogeneous for pooling a bivariate meta-analysis was performed. This approach takes into account the fact that even in a binary test there is an implicit cut-off point for abnormality. Varying this cut-off point would impact sensitivity and specificity in opposite directions, such that raising the threshold for abnormality would reduce the sensitivity and increase the specificity. The bivariate model incorporates this negative correlation between sensitivity and specificity (Doebler et al., 2025). The index test was clinical EEG result, and the reference standard was a considered clinical diagnosis in the opinion of the report authors. Descriptive statistics were calculated and tabulated. Between-study statistical heterogeneity was explored using the *I*^*2*^ statistic. The analysis was conducted using *R* version 4.3.1 and the *mada* package version 0.5.8 (Comprehensive R Archive Network, n.d.). Statistical significance was set at <0.05. A 0.5 continuity correction was applied due to the presence of zero cells in the data set (Pambabay et al., 2020). However, studies where there were no data to contribute to calculations of either sensitivity or specificity were excluded from calculations of the area under the receiver operator characteristics curve (AUC) and the summary receiver operating characteristics (SROC) curve. The primary outcome was whether an EEG was reported as abnormal. In this paper, an abnormal EEG is considered a positive finding, while a normal EEG is considered a negative finding. A true positive result would be a patient with psychosis with a secondary cause, who had an abnormal EEG. Secondary outcome measures were specific EEG abnormalities. The main measures of effect were sensitivity and specificity with 95 % confidence intervals. Positive predictive value and negative predictive value were calculated using a meta-analytic estimate of the prevalence of secondary psychosis of 11 % (95 % CI 7–16 %) among all cases of psychosis (Blackman et al., 2024). A Fagan’s nomogram was produced to support clinical interpretation.

In the first set of sensitivity analyses, the main analysis was repeated excluding certain studies that may introduce bias, specifically studies where there were no data on which to calculate sensitivity or specificity, studies including patients on any psychotropic medication, studies including patients on any antipsychotic medication, studies including patients on clozapine, studies including patients with past neurological disorders, studies rated at high or unclear risk of bias on the index test and reference standard sections of the QUADAS-2 tool, studies published before 2010, studies where 90 - <100 % of participants had psychosis, and studies where patients were selected based on being atypical in some way or were lacking consecutive enrolment. Papers published before 2010 were excluded in a sensitivity analysis due to the identification of the autoimmune disease anti-*N*-methyl-D-aspartate receptor encephalitis (ANDMRE) in 2007 (Pambabay et al., 2020). By 2010, clinicians were more aware of this phenomenon, which might have been previously unrecognised or misreported. Due to the risk for a 0.5 continuity correction to introduce a bias towards the null hypothesis in meta-analyses of diagnostic test accuracy (Mitchell, 2003), we conducted a second set of sensitivity analyses in which we used continuity corrections of different sizes (0.1, 0.01, 10^−4^, 10^−8^) (Sweeting M et al., 2004).

Subgroup analyses were conducted by specific EEG abnormality. Due to the large impact of clozapine use on the result, these subgroup analyses were repeated excluding studies where patients may have been taking clozapine. Subgroup analyses were also conducted by study type (cohort study vs case series). The full code used is available at https://osf.io/pj6nc/.

## 3 Results

### 3.1 Search results and overall EEG results

**Fig. 1:**
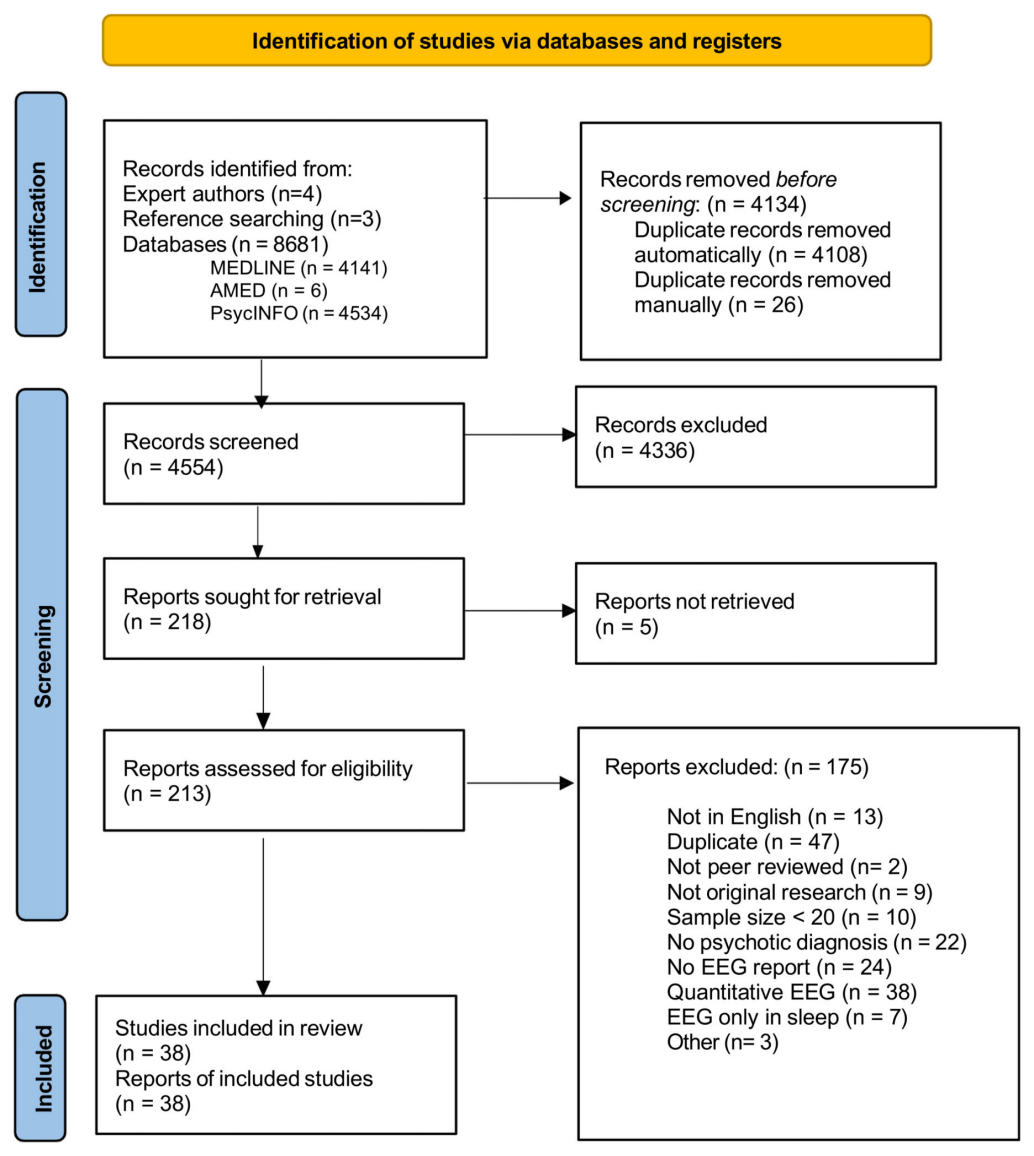
PRISMA flow chart.

The search strategy yielded 8688 results, which after deduplication left 4554 articles. Screening and eligibility assessment resulted in 38 studies included for meta-analysis (Fig. 1) (Gschwandtner et al., 2009; Restrepo-Martinez et al., 2020; Hinotsu et al., 2022; Endres et al., 2016; Endres et al., 2020; Kim et al., 2019b; Kroc et al., 2018; Chaychi et al., 2015; Okruszek et al., 2014; Raybould et al., 2012; Degner et al., 2011; Wichniak et al., 2006; Röttig et al., 2005; Pogarell et al., 2004; Amann et al., 2003; Schmitz et al., 1999; Inui et al., 1998; Freudenreich et al., 1997; Neufeld et al., 1996; Kanemoto et al., 1996; Olesen et al., 1995; Haring et al., 1994; Green et al., 1992; Sandyk and Kay, 1992; Nagakubo et al., 1991; Koufen and Hagel, 1987; Kendler and Hays, 1982; Guasp et al., 2021; Kikuchi et al., 2014; Shrivastava et al., 2014; Price et al., 2002; Sandyk and Awerbuch, 1993; Psatta et al., 1991; Wong et al., 1997; Welch et al., 1994; Small et al., 1984; Jefsen et al., 2023; Tsutsui et al., 2012).

Characteristics of included studies are presented in Table 1. There were six cohort studies and 32 case-series. In total, 3784 patients were included with a mean age of 36·1 years, with studies ranging from a mean (SD) of 9·6 (1·6) years to 73 (9·7) years. Data on biological sex was available for 3553 people, of these 1731 were males (45·7 %) and 1822 females (48·2 %), while sex was not reported in 231 patients (6·1 %). No included patients were reported to be non-binary or transgender. The index test for all studies was clinical scalp EEG.

1. (psychosis or psychotic or schizo* or delusion* or hebephren* orparaphren* or hallucinat* or mania or manic or bipolar disorder or bipolar affective).mp. [mp=ab, hw, ti, bt, ot, nm, fx, kf, ox, px, rx, ui, sy, ux, mx, tc, id, tm, mf]
2. exp Psychotic Disorders/
3. exp ”Schizophrenia Spectrum and Other Psychotic Disorders"/ or Schizophrenia/
4. exp Delusions/
5. exp Hallucinations/
6. exp Bipolar Disorder/ or exp Mania/
7. (eeg or electroencephalogr* or electrocerebral or telemetr*).mp. [mp=ab, hw, ti, bt, ot, nm, fx, kf, ox, px, rx, ui, sy, ux, mx, tc, id, tm, mf]
8. exp Electroencephalography/
9. 1 or 2 or 3 or 4 or 5 or 6
10. 7 or 8
11. 9 and 10
12. limit 11 to (humans and yr="1980 -Current")
13. exp "Review"/ or exp "Systematic Review"/
14. exp Case Reports/
15. 13 or 14
16. 12 not 15

In terms of diagnostic groups, 364 (9·6 %) patients had a final diagnosis of secondary psychosis (of whom 319 had an abnormal EEG), with the remaining 3420 (90·4 %) diagnosed with a primary psychiatric disorder (of whom 1090 had an abnormal EEG). In studies that reported specific EEG abnormalities in primary psychosis the most frequently reported were encephalopathy (17.2 %), epileptiform discharges (5.8 %), and non-epileptiform focal changes (2.5 %). In studies that reported specific EEG abnormalities in secondary psychosis the most frequently reported were encephalopathy (55.6 %), epileptiform discharges (34.0 %), and non-epileptiform focal changes (31.3 %) (Table 2).

**Fig. 2:**
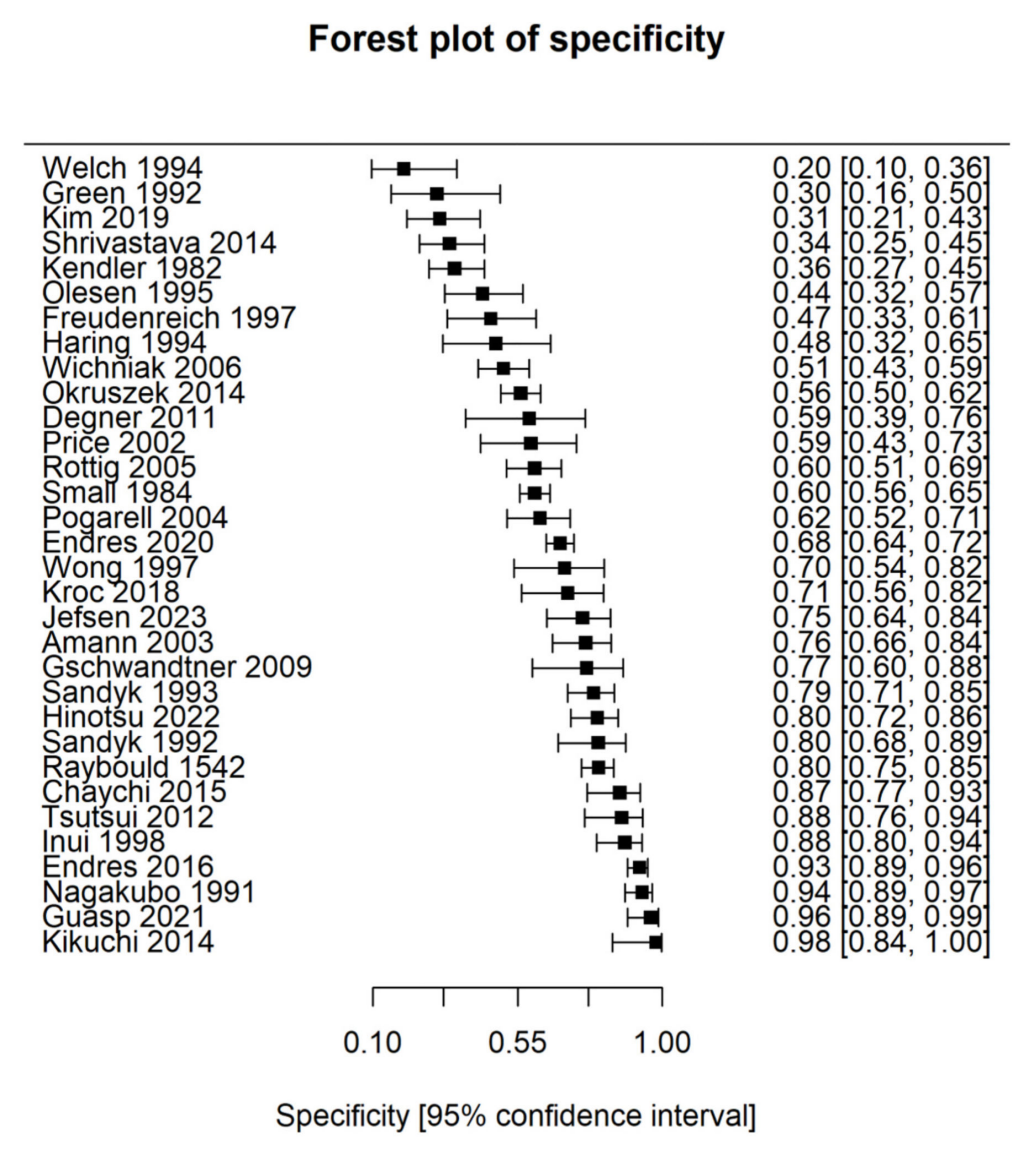
Forest plots showing specificity of included studies. Forest plot displaying the specificity (true negative rate) of each study assessing clinical EEG for identifying secondary psychosis, where an estimate was available. Squares represent point estimates of specificity, and horizontal whiskers indicate the corresponding 95 % confidence intervals. Studies are ordered in ascending order of specificity point estimates.

**Fig. 3:**
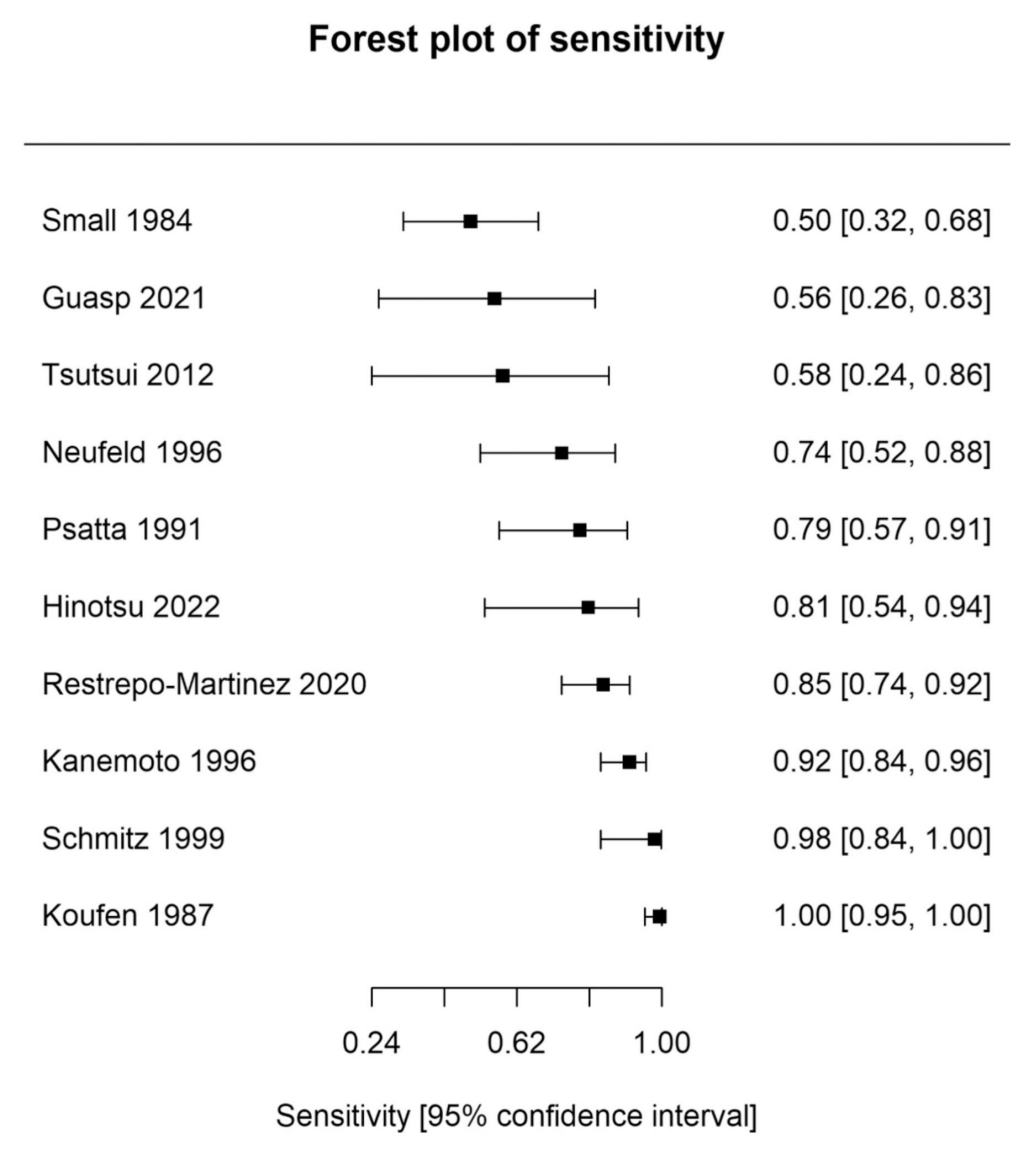
Forest plots showing sensitivity of included studies. Forest plot displaying the sensitivity (true positive rate) of each study assessing clinical EEG for identifying secondary psychosis, where an estimate was available. Squares represent point estimates of sensitivity, and horizontal whiskers indicate the corresponding 95 % confidence intervals. Studies are ordered in ascending order of specificity point estimates.

The diagnostic test results for individual studies are displayed in Table 3. Figs. 2 and 3 display a forest plot for the sensitivity and specificity of individual studies; no summary statistics are provided because the meta-analytic method synthesises the results of the sensitivity and specificity together. Of note, 28 studies included only patients with primary psychosis, so sensitivity could not be derived from these studies. Six studies included only patients with secondary psychosis, so specificity could not be derived from these studies. Diagnostic odds ratios and likelihood ratios for the individual studies can be found in Supplementary Table 3.

### 3.2 Main analysis

**Fig. 4:**
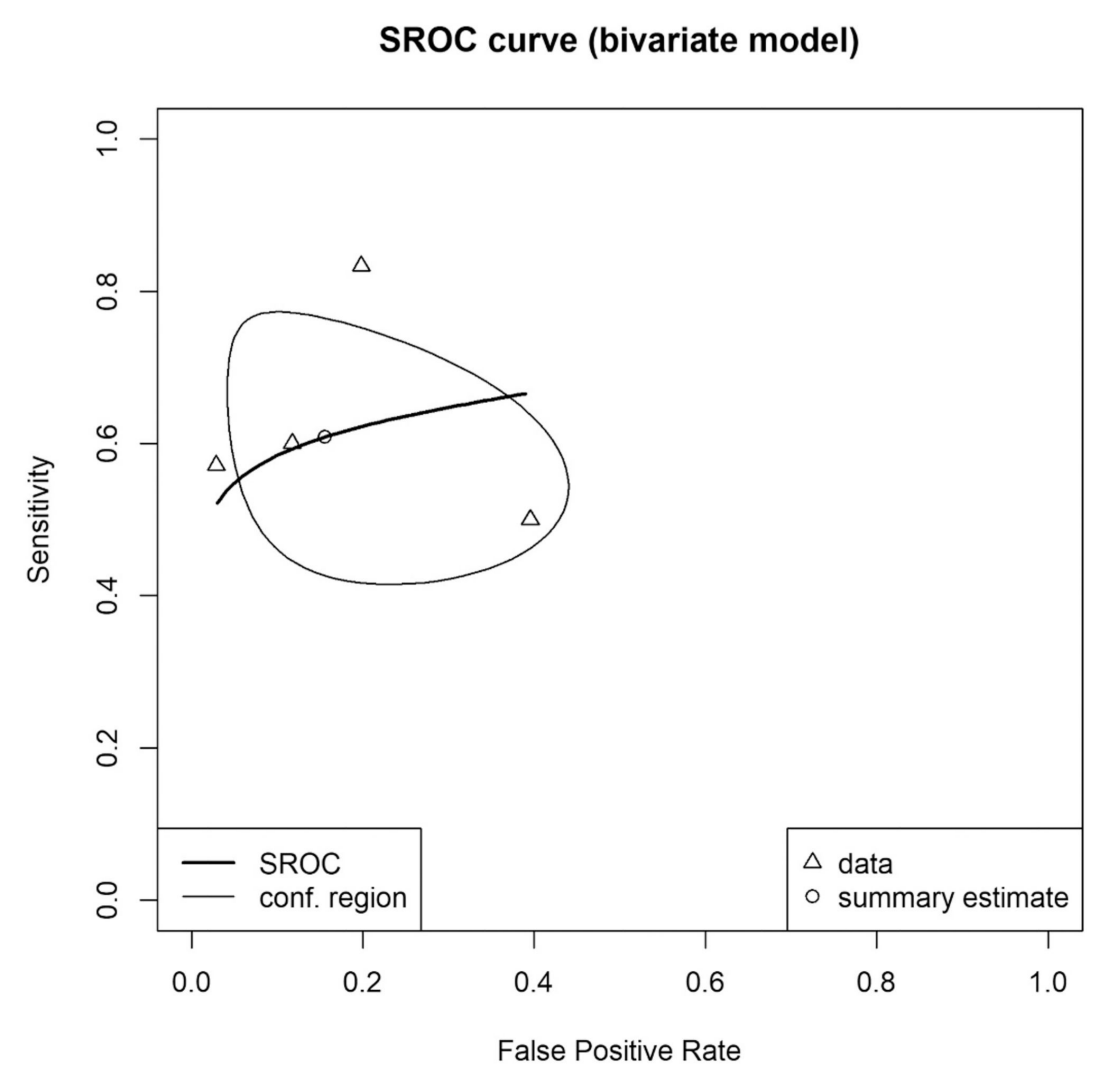
Summary receiver operating curve. Summary receiver operating curve (SROC) for studies were estimates for sensitivity and specificity could be derived. Individual study operating points are plotted as circles. The solid curve is the fitted SROC; the solid diamond marks the summary operating point. Surrounding this point the solid ellipse is the 95 % confidence region.

The main diagnostic test accuracy meta-analysis found that the sensitivity (i.e., the proportion of patients with secondary psychosis who had an abnormal EEG) was 0·71 (95 % CI 0·61–0·80; *I*^*2*^ = 9·2 %) and the specificity (i.e., the proportion of patients with primary psychosis who had a normal EEG) was 0·67 (95 % CI 0·58–0·74; *I*^*2*^ = 9·2 %). The proportion of variance accounted for by between-study heterogeneity was relatively low (*I*^*2*^ = 9·2 %). A summary receiver operating characteristics (SROC) curve is shown in Fig. 4 with an area under the SROC curve of 0·67, corresponding to poor discrimination (Mitchell, 2003). The positive likelihood ratio was 2·16 (95 % CI 1·65–2·80) and the negative likelihood ratio was 0·44 (95 % CI 0·30–0·59). The diagnostic odds ratio was 5·17 (95 % CI 2·86–8·66). Assuming a pretest probability of secondary psychosis of 11 % (95 % CI 7–16 %) (the prevalence of secondary psychosis among all cases of psychosis according to a recent meta-analysis) (Blackman et al., 2024), the positive predictive value of the EEG is 0·21 (range dependent on confidence intervals of prevalence 0·14–0·29) and the negative predictive value is 0·95 (0·92–0·97). Fagan's nomogram is presented in supplementary Fig. 1.

### 3.3 Risk of bias and applicability

Assessment for risk of bias and applicability with QUADAS-2 reported that 8 of the 38 studies were considered at high or unclear risk of bias in the patient flow measure and the remaining 30 were at low risk of bias, 17 were considered at high or unclear risk of bias and 21 were considered at low risk of bias for the patient selection measure, 16 were considered at high or unclear risk of bias and 22 were considered low risk of bias for the reference standard measure and 21 were considered at high or unclear risk of bias for the index test measure with the remaining 17 considered at low risk of bias for this parameter (Supplementary Fig. 2).

### 3.4 Subgroup and sensitivity analyses

A subgroup analysis was conducted for specific EEG abnormalities and can be seen in Table 3. The pattern for each of the EEG abnormalities was a low-moderate sensitivity and high specificity in identifying secondary psychosis.

Results of the sensitivity analyses excluding certain studies are shown in Table 4. In some analyses, it was impossible to estimate pooled sensitivity because of an absence of secondary psychosis cases. A sensitivity analysis of studies reporting only consecutively recruited patients with a psychotic presentation evidenced a sensitivity of 0·69, specificity of 0·70 and an AUC of 0·93. Furthermore, restricting to only studies that reported a consecutive cohort of patients presenting with undifferentiated psychosis evidenced a sensitivity of 0·52, specificity of 0·82 and an AUC of 0·93. Where other estimates were available, they were generally similar to the main analysis.

Result of the sensitivity analyses in which the continuity corrections were varied are shown in Supplementary Table 7. The sensitivity varied between 0·71 and 0·78 and the specificity varied between 0·67 and 0·68.

Results for subgroup analysis of only studies of cohort design and only studies of case series design are reported in supplementary table 8. Including only studies of cohort design resulted in a sensitivity of 0·69, specificity of 0·76 and AUC of 0·57. Including only studies of case series design resulted in a sensitivity of 0·76, specificity of 0·65 and AUC of 0·86.

## 4 Discussion

In what we believe to be the first systematic review and meta-analysis of the diagnostic test accuracy of EEG in psychoses of varying aetiology, including 38 studies and 3784 patients, we found that scalp EEG has poor discrimination in ascertaining whether psychosis is due to a secondary cause (Šimundić, 2009). The main diagnostic test accuracy meta-analysis found that the sensitivity (i.e., the proportion of patients with secondary psychosis who had an abnormal EEG) was 0·71 (95 % CI 0·61–0·80) and the specificity (i.e., the proportion of patients with primary psychosis who had a normal EEG) was 0·67 (95 % CI 0·58–0·74).

The results of the meta-analysis do not show particularly high specificity. One explanation may be related to the significant challenge in using EEG in populations of people with a psychotic disorder because of the impact of antipsychotic medication. Studies have found that antipsychotics, particularly clozapine, can result in EEG abnormalities such as generalized slowing and epileptiform discharges (Jackson and Seneviratne, 2019). The initiation of clozapine increased the odds of epileptiform discharges by six (OR 6·2 (95 % 3·14–11·3) and EEG slowing by almost 17 times (OR 16·9 (95 % CI 5·4 to 53·2) (Jackson and Seneviratne, 2019). Expectedly only two of the studies reported results for individuals with primary psychosis who were antipsychotic-naïve, so this may have affected the EEG abnormality rates in the population and therefore influenced the specificity findings in the current study. To explore this, we conducted sensitivity analyses excluding individuals who were on clozapine and secondly, excluding individuals on any antipsychotic (Table 4). The results showed a higher specificity in both the clozapine-excluded (0·75 (95 % CI 0·58–0·87)) and antipsychotic-excluded analyses (0·82 (95 % CI 0·33–0·97)). However, it is important to note the small number of studies included in the sensitivity analyses (k = 7 and 2 respectively) and confidence intervals were broad.

The current study showed that breaking down EEG analysis into specific abnormalities, as seen in Table 2, may yield more valuable insights into the aetiology of a psychotic disorder. EEG findings of asymmetry, non-epileptiform focal changes, rhythmic changes, features suggestive of limbic encephalitis, non-convulsive status epilepticus, epileptiform discharges, and periodic activity revealed low sensitivity and high specificity. These results suggest the presence of these specific findings in patients presenting with psychosis are useful for supporting a secondary diagnosis, but further investigation may be necessary in their absence. NMDAR encephalitis may present with psychosis and the most common EEG abnormalities in this condition include encephalopathy, delta range slowing, focal abnormalities and epileptiform activity, while the extreme delta brush is highly specific (Gillinder et al., 2019; Schmitt et al., 2012). EEG findings in post ictal psychosis are heterogeneous with diffuse slow wave and spike and sharp wave patterns evidenced (Cheval et al., 2024).

A specific EEG finding of encephalopathy was the most reported abnormality in patients with primary psychosis (*n* = 408) and evidenced a higher sensitivity (0·51 (95 % CI 0·34–0·67)), but reduced specificity (0·87 (95 % CI 0·78–0·93)) compared with the other specific EEG abnormalities, suggesting that a encephalopathic findings on an EEG in psychosis regularly may a false positive and should be interpreted in the context of prior probability, other investigations, and prescribed medications.

Strengths of this study include its large sample size and its varied source population across a variety of clinical settings and 16 different countries. Limitations of the study can be categorised in terms of the included studies and of the synthesis itself. Of the included studies, 21 were considered at high or unclear risk of bias for the index test measure (EEG). This was explored with a sensitivity analysis excluding papers with a high or unclear risk of index test bias. As seen in Table 4, the analysis of 17 studies with a low risk of index test bias showed a similar specificity to the main analysis (0·65 (95 % CI 0·54–0·74)), and a sensitivity of 0·50 (95 % CI 0·36–0·65) which was derived from a single study. Another limitation is the impact of the reference standard. In the current study, the reference standard of 16 papers was at high or unclear risk of bias, with the results from the sensitivity analysis of papers at low risk of bias showing a higher AUC (0.88 vs 0.67). This may be related to the lack of comprehensive diagnostic tests used in some studies to delineate secondary from primary psychosis; future studies should ensure an adequate array of diagnostic tests are performed to reduce the risk of misclassification bias. This reflects a more core issue in psychosis research and diagnosis. In psychosis, and psychiatric disorders more widely, there is an issue of our current diagnostic paradigms, such as DSM-5 or ICD-11 (American Psychiatric Association, 2013; World Health Organization, 2022), relying solely on signs and symptoms that may have an imprecise correlation with underlying neurobiology (Tsutsui et al., 2012). As in our study, a diagnosis of psychosis is often based on a considered clinical opinion. This poses challenges in terms of the validity and consistency of assessment, as clinical opinions can vary significantly based on various factors. There are also no consensus recommendations on which, and how many, investigations should be performed to identify cases of secondary psychosis. When the analysis was restricted to only consecutively recruited patients presenting with undifferentiated psychosis, EEG evidenced a sensitivity of 0.52, specificity of 0.82 and AUC of 0.93 suggesting that EEG may represent a useful rule out test for secondary psychosis in this context. Although given the small number of studies that met these criteria (k = 4) further research is required to elucidate these findings.

In terms of limitations of the synthesis itself, an important consideration is that there was a small sample of secondary cases. Of the individuals in our combined data set, 364 had a final diagnosis of secondary psychosis. This has a potential effect on the results in terms of sensitivity, although not specificity. Secondly, the absence of data on secondary psychosis cases meant that building the SROC was based on a small number of studies. Our bivariate meta-analysis model used a 0.5 continuity correction, which may have introduced a bias towards the null hypothesis. We note that reducing the size of the continuity correction slightly raised the estimated sensitivity of EEG to 0.78 (0.65–0.87); and this estimate remains imprecise and is based on only a small number of studies. As a result, these model-based findings should be interpreted with caution, since the pooled sensitivity may change should additional studies reporting both sensitivity and specificity become available. However, there would not be a valid imputation strategy to address these zero cells, given that many studies had no secondary psychosis patients. Thirdly, we note that while the main meta-analysis across many different EEG abnormalities indicates poor discrimination in establishing primary or secondary psychosis, specificity was much higher when examining EEGs at the level of specific abnormalities. Finally, it is important to recognise that the EEG is a dynamic modality which may evolve over time and repeat testing may be indicated in cases of diagnostic uncertainty.

With regard to implications for future research, our experience of extracting EEG data showed that reporting of EEGs in the included studies was inconsistent and lacking in detail, merely reporting an EEG as normal or abnormal. We therefore reiterate calls for a minimum EEG reporting standard for published reports, in line with the American Clinical Neurophysiology Society's guidance (Hosseini et al., 2023; Tatum et al., 2016). Furthermore, the current study highlights the importance of considering specific abnormalities. Studies have highlighted the link between individual EEG abnormalities in certain psychiatric disorders, such as dysrhythmias and generalized slowing in patients with schizophrenia (Boutros et al., 2011). In-depth exploration of individual EEG abnormalities in psychosis may hold some valuable insights in terms of aetiology, prognosis, and treatment response (Lee and Kim, 2022; Tebartz van Elst et al., 2011). Further research is needed to explore how specific EEG abnormalities relate to this, potentially excluding individuals who are on antipsychotic medication.

Based on the findings of the current study, interpreting an EEG at the level of normal or abnormal shows poor discrimination. The moderate sensitivity and specificity indicate that it is crucial to interpret both a negative and positive EEG in psychosis in the context of presenting symptoms, physical examination and other investigations (Griswold et al., 2015). However, using specific EEG abnormalities potentially has value in determining a secondary cause of psychosis. Therefore, clinicians should consider the use of EEG in individuals presenting with a psychotic disorder of unknown aetiology. Examples of ‘red flags’ that might prompt such investigation include new-onset severe headache, adverse or insufficient response to antipsychotics, movement disorders (e.g., catatonia or dyskinesia), decreased consciousness, and comorbid autoimmune disorders (e.g., autoimmune thyroid disease) (Pollak et al., 2020). Clinicians should consider the specific EEG abnormalities, rather than merely whether an EEG is abnormal when using EEG as part of a diagnostic work-up.

## Supplementary Material

Supplementary data to this article can be found online at https://doi.org/10.1016/j.schres.2025.07.020.

Supplementary materials

## Figures and Tables

**Table 1:** Characteristics of included studies.

Study	Country	Study design	Sample size	Age (Years) (Median (SD) unless stated)	Sex	Reference standard	Clinical Setting
Hinotsu et al. (2022)	Japan	Case Series	123	36.3 (17.2)	Male: 36 Female: 87	DSM-5	Medical Hospital
Endres et al. (2020)	Germany	Case Series	449	35.3 (14.9)	Male: 207 Female: 242	ICD-10	Psychiatric Hospital
Restrepo-Martinez et al. (2020)	Mexico	Case Series	61	27.2 (8.4)	Male: 31 Female: 30	DSM-5	Neurological Hospital
Kim et al. (2019a, b)	South Korea	Case Series	59	37.7 (11.5)	Male: 33 Female: 26	DSM-IV	Psychiatric Hospital
Kroc et al. (2018)	Poland	Cohort	45	30.1 (8.7)	Male: 18 Female: 27	ICD-10	Psychiatric Hospital
Endres et al. (2016)	Germany	Case Series	251	37.8 (12.2)	Male: 108 Female: 143	NS	Psychiatric Hospital
Chaychi et al. (2015)	Iran	Case series	64	NS	Male: NS Female: NS	DSM-IV-TR	Psychiatric Hospital
Okruszek et al. (2014)	Poland	Cohort	244	28.2 (7.0)	Male: 144 Female: 100	ICD-10	Psychiatric Hospital
Raybould et al. (2012)	USA	Case series	240	38.7 (14.3)	Male: 122 Female: 118	DSM-IV	Medical Hospital
Degner et al. (2011)	Germany	Case series	22	44.8 (15.2)	Male: 15 Female: 7	ICD-10	Psychiatric Hospital
Gschwandtner et al.(2009)	Switzerland	Cohort	31	31.7 (7.3)	Male: 22 Female: 9	Basel Screening instrument for psychosis	Outpatient Clinic
Wichniak et al. (2006)	Poland	Case series	146	36.8 (7.8)	Male: 68 Female: 78	DSM-IV	Outpatient Clinic
Röttig et al. (2005)	Germany	Case series	119	41.2 (11.6)	Male: 26 Female: 93	ICD-10	Psychiatric Hospital
Pogarell et al. (2004)	Germany	Case series	90	36.7 (15.1)	Male: 51 Female: 39	ICD-10	Psychiatric Hospital
Amann et al. (2003)	Germany	Case series	81	36.7 (14.1)	Male: 43 Female: 38	DSM-IV	Psychiatric Hospital
Schmitz et al. (1999)	United Kingdom	Cohort	25	23.9 (10.6)	Male: 16 Female: 9	DSM-IV	Neurological Hospital
Inui et al. (1998)	Japan	Case series	81	34.5 (13.1)	Male: 29 Female: 52	DSM-IV	Psychiatric Hospital
Freudenreich et al.(1997)	USA	Case series	45	38 (range 21–56)	Male: 35 Female: 10	DSM-III-R	Psychiatric Hospital
Neufeld et al. (1996)	Israel	Case series	20	73 (9.7)	Male: 10 Female: 10	NS	NS
Kanemoto et al. (1996)	Japan	Case series	88	35.3 (11.7)	Male: 52 Female: 36	Clinical expertise	NS
Olesen et al. (1995)	Denmark	Case series	64	37.6 (9.1)	Male: 44 Female: 20	ICD-8	Mixed: Outpatient Clinic and Psychiatric Hospital
Haring et al. (1994)	Austria	Case series	29	31.7 (10.2)	Male: 18 Female: 11	DSM-III-R	NS
Green et al. (1992)	USA	Case series	24	9.6 (1.4)	Male: 16 Female: 8	DSM-III	Psychiatric Hospital
Sandyk and Kay (1992)	USA	Case series	52	51.3 (9.1)	Male: 29 Female: 23	DSM-III	Psychiatric Hospital
Nagakubo et al. (1991)	Japan	Cohort	137	NS	Male: NS Female: NS	NS	Outpatient Clinic
Koufen and Hagel (1987)	Germany	Case Series	100	Range 14–61	Male: 94 Female: 6	NS	NS
Kendler and Hays (1982)	Canada	Case series	113	NS	Male: 55 Female: 58	DSM-III	NS
Guasp et al. (2021)	Spain	Case series	76	30 (15.3)	Male: 43 Female: 33	NS	Psychiatric Hospital
Kikuchi et al. (2014)	Japan	Case series	26	36.6 (11.7)	Male: 8 Female 18:	NS	Psychiatric Hospital
Shrivastava et al. (2014)	Canada	Case series	80	33.4 (7.1)	Male: 66 Female: 14	NS	Psychiatric Hospital
Price et al. (2002)	Australia	Case Series	37	39.6 (12.3)	Male: NS Female: NS	DSM-IV	Psychiatric Hospital
Sandyk and Awerbuch (1993)	USA	Case Series	119	46.5 (16.4)	Male: 22 Female: 97	DSM-III	Psychiatric Hospital
Psatta et al. (1991)	Romania	Case series	20	Range 20–52	Male: 8 Female: 12	DSM-III	Outpatient Clinic
Wong et al. (1997)	United Kingdom	Case series	37	39.4 (9.8)	Male: 37 Female: 0	NS	Psychiatric Hospital
Welch et al. (1994)	USA	Case series	32	41.3 (10.8)	Male: 14 Female: 18	DSM-IIIR	Psychiatric Hospital
Small et al. (1984)	USA	Cohort	433	35.6	Male: 165 Female: 268	DSM-II	Psychiatric Hospital
Jefsen et al. (2023)	Denmark	Case series	70	32	Male: 35 Female: 35	ICD-10	Psychiatric Hospital
Tsutsui et al. (2012)	Japan	Case series	66	Range 15–61	Male: 12 Female: 44	ICD-10	Medical Hospital

Key: EEG = electroencephalogram, NS = not specified, ICD-8 = International Classification of Diseases 8th Revision, ICD-10 = International Classification of Diseases 10th Revision, DSM-III = Diagnostic and Statistical Manual of Mental Disorders 3rd Edition, DSM-III-R = Diagnostic and Statistical Manual of Mental Disorders 3rd Edition Revised; DSM-IV = Diagnostic and Statistical Manual of Mental Disorders 4rd Edition, DSM-IV-TR = Diagnostic and Statistical Manual of Mental Disorders 4th Edition Text Revision, DSM-5 = Diagnostic and Statistical Manual of Mental Disorders 5th Edition. (see supplementary materials for citations).

**Table 2:** Diagnostic accuracy of specific EEG abnormalities.

*Abnormality*	No. of studies	No. of primary psychosis patients (patients with abnormality)	No. of secondary psychosis patients (patients with abnormality)	Sensitivity (95 %CI)	Specificity (95 %CI)	AUC	I^2^
*Any abnormality (main analysis)*	38	3420 (1090)	364 (319)	0·71 (0·61–0·80)	0·67 (0·58–0·74)	0·67	9 %
*Encephalopathy*	30	2379 (408)	338 (188)	0·51 (0·34–0·67)	0·87 (0·78–0·93)	0·88	11 %
*Asymmetry*	30	2379 (34)	338 (8)	0·21 (0·11–0·35)	0·98 (0·95–0·99)	0·89	0 %
*Non epileptiform Focal changes*	30	2379 (59)	338 (106)	0·32 (0·17–0·51)	0·97 (0·94–0·98)	0·99	7 %
*Rhythmic changes*	30	2379 (18)	338 (0)	0·19 (0·09–0·35)	0·98 (0·96–0·99)	0·89	0 %
*Features suggestive of limbic encephalitis*	30	2379 (0)	338 (9)	0·22 (0·11–0·37)	0·99 (0·97–0·99)	0·89	0 %
*Epileptiform discharge*	30	2379 (139)	338 (115)	0·37 (0·22–0·54)	0·93 (0·90–0·96)	0·46	8 %
*Non convulsive status epilepticus*	30	2379 (0)	338 (2)	0·20 (0·10–0·36)	0·99 (0·97–0·99)	0·89	0 %

**Table 3:** 2 × 2 results of individual studies.

Study	True positives (secondary psychosis with an abnormal EEG)	False positives (primary psychosis with an abnormal EEG)	False negatives (secondary psychosis with a normal EEG)	True negatives (primary psychosis with a normal EEG)
Hinotsu et al. (2022)	10	22	2	89
Endres et al. (2020)	0	142	0	307
Restrepo-Martinez et al. (2020)	52	0	9	0
Kim et al. (2019a, b)	0	41	0	18
Kroc et al. (2018)	0	13	0	32
Endres et al. (2016)	0	17	0	234
Chaychi et al. (2015)	0	8	0	56
Okruszek et al. (2014)	0	107	0	137
Raybould et al. (2012)	0	47	0	193
Degner et al. (2011)	0	9	0	13
Gschwandtner et al. (2009)	0	7	0	24
Wichniak et al. (2006)	0	72	0	74
Röttig et al. (2005)	0	47	0	72
Pogarell et al. (2004)	0	34	0	56
Amann et al. (2003)	0	19	0	62
Schmitz et al. (1999)	25	0	0	0
Inui et al. (1998)	0	9	72	0
Freudenreich et al. (1997)	0	24	0	21
Neufeld et al. (1996)	15	0	5	0
Kanemoto et al. (1996)	81	0	7	0
Olesen et al. (1995)	0	33	5	26
Haring et al. (1994)	0	15	0	14
Green et al. (1992)	0	17	0	7
Sandyk and Kay (1992)	0	10	0	42
Nagakubo et al. (1991)	0	8	0	129
Koufen and Hagel (1987)	100	0	0	0
Kendler and Hays (1982)	0	73	0	40
Guasp et al. (2021)	4	2	3	67
Kikuchi et al. (2014)	0	0	0	26
Shrivastava et al. (2014)	0	53	0	27
Price et al. (2002)	0	15	0	22
Sandyk and Awerbuch (1993)	0	25	0	94
Psatta et al. (1991)	16	0	4	0
Wong et al. (1997)	0	11	0	26
Welch et al. (1994)	0	26	0	6
Small et al. (1984)	13	161	13	246
Jefsen et al. (2023)	0	17	0	53
Tsutsui et al. (2012)	3	6	2	55

**Table 4:** Diagnostic accuracy of sensitivity analyses.

*Sensitivity analysis*	No. of studies	No. of patients (patients with abnormality)	Sensitivity (95 % CI)*	Specificity (95 % CI)	AUC*	I^2^
*Main analysis*	38	3784 (1409)	0·71 (0·61–0·80)	0·67 (0·58–0·74)	0·67	9 %
*Excluding studies before 2010*	15	1866 (553)	0·73 (0·59–0·83)	0·75 (0·61–0·85)	0·86	0 %
*Excluding patients taking any psychotropics*	1	37 (15)	· ·	0·59 (0·43–0·73)	· ·	60 %
*Excluding patients taking any antipsychotics*	2	288 (32)	· ·	0·82 (0·34–0·98)	· ·	82 %
*Excluding patients taking clozapine*	7	676 (170)	· ·	0·75 (0·58–0·87)	· ·	48 %
*Excluding studies with patients with past neurological disorders*	9	1038 (305)	· ·	0·72 (0·55–0·84)	· ·	59 %
*Excluding studies at high risk of index test bias*	17	2200 (782)	0·50 (0·35–0·65)	0·65 (0·54–0·74)	0.57	23 %
*Excluding studies at high risk of reference test bias*	22	2086 (880)	0·75 (0·63–0·84)	0·61 (0·48–0·72)	0.88	17 %
*Excluding studies that lack data on either sensitivity or specificity*	4	688 (221)	0·60 (0·45–0·73)	0·84 (0·63–0·94)	0·67	0 %
*Excluding studies that reported any patients with a non-psychotic presentation*	33	3285 (1229)	0·69 (0·57–0·79)	0·66 (0·57–0·74)	0·59	10 %
*Excluding studies where consecutive recruitment of typical patients was not specified*	4	796 (202)	0.52 (0.25–0.78)	0.82 (0.64–0.92)	0.93	0 %

* Sensitivity and AUC could not be calculated for studies where there were no patients with secondary psychosis.

## Data Availability

The data used in this systematic review and meta-analysis is available upon reasonable request to the corresponding author.
