# Supplementary material for: The role of the electroencephalogram (EEG) in determining the aetiology of psychosis: a systematic review and meta-analysis of diagnostic test accuracy
Source: Schizophr Res. Author manuscript; Available in PMC 2026 Feb 19. (PMC7618760; doi:10.1016/j.schres.2025.07.020)

# Supplementary Materials

**Full search strategy**

1. (psychosis or psychotic or schizo* or delusion* or hebephren* or paraphren* or hallucinat* or mania or manic or bipolar disorder or bipolar affective).mp. [mp=ab, hw, ti, bt, ot, nm, fx, kf, ox, px, rx, ui, sy, ux, mx, tc, id, tm, mf]
2. exp Psychotic Disorders/
3. exp "Schizophrenia Spectrum and Other Psychotic Disorders"/ or Schizophrenia/
4. exp Delusions/
5. exp Hallucinations/
6. exp Bipolar Disorder/ or exp Mania/
7. (eeg or electroencephalogr* or electrocerebral or telemetr*).mp. [mp=ab, hw, ti, bt, ot, nm, fx, kf, ox, px, rx, ui, sy, ux, mx, tc, id, tm, mf]
8. exp Electroencephalography/
9. 1 or 2 or 3 or 4 or 5 or 6
10. 7 or 8
11. 9 and 10
12. limit 11 to (humans and yr="1980 -Current")
13. exp "Review"/ or exp "Systematic Review"/
14. exp Case Reports/
15. 13 or 14
16. 12 not 15
17. 16 use medall
18. (psychosis or psychotic or schizo* or delusion* or hebephren* or paraphren* or hallucinat* or mania or manic or bipolar disorder or bipolar affective).mp. [mp=ab, hw, ti, bt, ot, nm, fx, kf, ox, px, rx, ui, sy, ux, mx, tc, id, tm, mf]
19. exp Psychosis/
20. exp Schizophrenia/
21. exp Delusions/
22. exp Hallucinations/
23. exp Mania/
24. exp Bipolar Disorder/
25. (eeg or electroencephalogr* or electrocerebral or telemetr*).mp. [mp=ab, hw, ti, bt, ot, nm, fx, kf, ox, px, rx, ui, sy, ux, mx, tc, id, tm, mf]
26. exp Electroencephalography/
27. 18 or 19 or 20 or 21 or 22 or 23 or 24
28. 25 or 26
29. 27 and 28
30. limit 29 to (human and yr="1980 -Current")
31. exp "Literature Review"/ or exp "Systematic Review"/
32. exp Case Report/
33. 31 or 32
34. 30 not 33
35. 34 use psyh
36. (psychosis or psychotic or schizo* or delusion* or hebephren* or paraphren* or hallucinat* or mania or manic or bipolar disorder or bipolar affective).mp. [mp=ab, hw, ti, bt, ot, nm, fx, kf, ox, px, rx, ui, sy, ux, mx, tc, id, tm, mf]
37. exp Psychotic disorders/
38. exp Schizophrenia/
39. exp Delusions/
40. exp Hallucinations/
41. exp Bipolar disorder/
42. (eeg or electroencephalogr* or electrocerebral or telemetr*).mp. [mp=ab, hw, ti, bt, ot, nm, fx, kf, ox, px, rx, ui, sy, ux, mx, tc, id, tm, mf]
43. exp Electroencephalography/
44. 36 or 37 or 38 or 39 or 40 or 41
45. 42 or 43
46. 44 and 45
47. limit 46 to yr="1980 -Current"
48. exp Case report/
49. 47 not 48
50. 49 use amed
51. 17 or 35 or 50

References for **Table 1:**

*Key: ICD-8 = International Classification of Diseases 8th Revision*^1^*, ICD-10 = International Classification of Diseases 10th Revision*^2^*, DSM-III = Diagnostic and Statistical Manual of Mental Disorders 3^rd^ Edition*^3^*, DSM-III-R = Diagnostic and Statistical Manual of Mental Disorders 3^rd^ Edition Revised*^4^, *DSM-IV = Diagnostic and Statistical Manual of Mental Disorders 4^th^ Edition*^5^*, DSM-IV-TR = Diagnostic and Statistical Manual of Mental Disorders 4^th^ Edition Text Revision*^6^*, DSM-5 = Diagnostic and Statistical Manual of Mental Disorders 5^th^ Edition*^7^*.*

1. World Health Organization: International statistical classification of diseases and related health problems (8th Revision).; 1968.

2. World Health Organization: International statistical classification of diseases and related health problems (10th Revision). 10th ed.; 2016.

3. American Psychiatric Association: Diagnostic and statistical manual of mental disorders (3rd ed.). 3rd ed.; 1980.

4. American Psychiatric Association: Diagnostic and statistical manual of mental disorders (3rd ed., rev.). 3rd ed.; 1987.

5. American Psychiatric Association: Diagnostic and statistical manual of mental disorders (4th ed.). 4th ed.; 1994.

6. American Psychiatric Association: Diagnostic and statistical manual of mental disorders (4th ed., text rev.). 4th ed.; 2000.

7. American Psychiatric Association: Diagnostic and statistical manual of mental disorders (5th ed.). 5th ed.; 2013.

**Supplementary Table 1:** PRISMA DTA Checklist

| **Section/topic** | **#** | **PRISMA-DTA Checklist Item** | **Reported on page #** |
| --- | --- | --- | --- |
| **TITLE / ABSTRACT** | | |  |
| Title | 1 | Identify the report as a systematic review (+/- meta- analysis) of diagnostic test accuracy (DTA) studies. | 1 |
| Abstract | 2 | Abstract: See PRISMA-DTA for abstracts. | 2 |
| **INTRODUCTION** | | |  |
| Rationale | 3 | Describe the rationale for the review in the context of what is already known. | 4 |
| Clinical role of index test | D1 | State the scientific and clinical background, including the intended use and clinical role of the index test, and if applicable, the rationale for minimally acceptable test accuracy (or minimum difference in accuracy for comparative design). | 4 |
| Objectives | 4 | Provide an explicit statement of question(s) being addressed in terms of participants, index test(s), and target condition(s). | 4 |
| **METHODS** | | |  |
| Protocol and registration | 5 | Indicate if a review protocol exists, if and where it can be accessed (e.g., Web address), and, if available, provide registration information including registration number. | 7 |
| Eligibility criteria | 6 | Specify study characteristics (participants, setting, index test(s), reference standard(s), target condition(s), and study design) and report characteristics (e.g., years considered, language, publication status) used as criteria for eligibility, giving rationale. | 6-7 |
| Information sources | 7 | Describe all information sources (e.g., databases with dates of coverage, contact with study authors to identify additional studies) in the search and date last searched. | 5-6 |
| Search | 8 | Present full search strategies for all electronic databases and other sources searched, including any limits used, such that they could be repeated. | 5-6 |
| Study selection | 9 | State the process for selecting studies (i.e., screening, eligibility, included in systematic review, and, if applicable, included in the meta-analysis). | 7 |
| Data collection | 10 | Describe method of data extraction from reports (e.g., piloted forms, independently, in duplicate) and any | 7 |

| process |  | processes for obtaining and confirming data from investigators. |  |
| --- | --- | --- | --- |
| Definitions for data extraction | 11 | Provide definitions used in data extraction and classifications of target condition(s), index test(s), reference standard(s) and other characteristics (e.g. study design, clinical setting). | 7  Supplementary table 2 |
| Risk of bias and applicability | 12 | Describe methods used for assessing risk of bias in individual studies and concerns regarding the applicability to the review question. | 7 |
| Diagnostic accuracy measures | 13 | State the principal diagnostic accuracy measure(s) reported (e.g. sensitivity, specificity) and state the unit of assessment (e.g. per-patient, per-lesion). | 7-8 |
| Synthesis of results | 14 | Describe methods of handling data, combining results of studies and describing variability between studies. This could include, but is not limited to: a) handling of multiple definitions of target condition. b) handling of multiple thresholds of test positivity, c) handling multiple index test readers, d) handling of indeterminate test results, e) grouping and comparing tests, f) handling of different reference standards | 7-8 |
| Meta-analysis | D2 | Report the statistical methods used for meta-analyses, if performed. | 7-8 |
| Additional analyses | 16 | Describe methods of additional analyses (e.g., sensitivity or subgroup analyses, meta-regression), if done, indicating which were pre-specified. | 7-8 |
| **RESULTS** |  |  |  |
| Study selection | 17 | Provide numbers of studies screened, assessed for eligibility, included in the review (and included in meta- analysis, if applicable) with reasons for exclusions at each stage, ideally with a flow diagram. | 9-10 |
| Study characteristics | 18 | For each included study provide citations and present key characteristics including: a) participant characteristics (presentation, prior testing), b) clinical setting, c) study design, d) target condition definition, e) index test, f) reference standard, g) sample size, h) funding sources | 9-10  Table 1 |
| Risk of bias and applicability | 19 | Present evaluation of risk of bias and concerns regarding applicability for each study. | 10 |
| Results of individual studies | 20 | For each analysis in each study (e.g. unique combination of index test, reference standard, and positivity threshold) report 2x2 data (TP, FP, FN, TN) with estimates of diagnostic accuracy and confidence intervals, ideally with a forest or receiver operator characteristic (ROC) plot. | Table 2 |
| Synthesis of results | 21 | Describe test accuracy, including variability; if meta- analysis was done, include results and confidence intervals. | 10  Figures 2, 3 and 4 |
| Additional analysis | 23 | Give results of additional analyses, if done (e.g., sensitivity or subgroup analyses, meta-regression; analysis of index test: failure rates, proportion of inconclusive results, adverse events). | 10  Tables 3 and 4 |
| **DISCUSSION** |  |  |  |

| Summary of evidence | 24 | Summarize the main findings including the strength of evidence. | 17-18 |
| --- | --- | --- | --- |
| Limitations | 25 | Discuss limitations from included studies (e.g. risk of bias and concerns regarding applicability) and from the review process (e.g. incomplete retrieval of identified research). | 17-18 |
| Conclusions | 26 | Provide a general interpretation of the results in the context of other evidence. Discuss implications for future research and clinical practice (e.g. the intended use and clinical role of the index test). | 18 |

*Adapted From:* McInnes MDF, Moher D, Thombs BD, McGrath TA, Bossuyt PM, The PRISMA-DTA Group (2018). Preferred Reporting Items for a Systematic Review and Meta-analysis of Diagnostic Test Accuracy Studies: The PRISMA-DTA Statement. JAMA. 2018 Jan 23;319(4):388-396. doi: 10.1001/jama.2017.19163.

**Supplementary Table 2:** Data extraction definitions

| **Extraction item** | **Definitions** |
| --- | --- |
| **Title** | Title of paper |
| **Authors** | All authors of the paper |
| **Abstract** | - |
| **Year** | Publication year |
| **Country** | Country of corresponding author |
| **Study design** | Exposure: neurological or general medical condition causing psychosis. Outcome: EEG   - Cohort study: individuals with a primary psychotic disorder AND individuals with a secondary psychotic disorder are followed up prospectively or retrospectively, then at a subsequent point in time an EEG is performed - Case-control study: psychotic individuals with normal and abnormal EEGs are taken, then researchers looked back in time to see what diagnoses they had been give - Case series: individuals with a primary psychotic disorder OR individuals with a secondary psychotic disorder (NOT both) are examined for their EEG findings - Cross-sectional study: at the same point in time, diagnoses of both primary & secondary psychotic disorders are made AND EEGs are performed |
| **Eligibility criteria** | Criteria for entry to the study, e.g. 'adults with a diagnosis of schizophrenia presenting to the Hans Berger Neuropsychiatric  Clinic without recreational drug use' |
| **Number of patients** | Number of subjects in the study that fit our criteria of having a psychotic disorder at time of EEG. |
| **Age (mean)** | Age in years at time of EEG. |
| **Age (SD)** | Age in years at time of EEG. |
| **Sex, male** | Number of subjects who were reported as male |
| **Sex, female** | Number of subjects who were reported as female |
| **Ethnicity** | - |
| **Number of subjects with past neuro disorder affecting the brain** | Number with a pre-existing neurological disorder affecting the brain, e.g. stroke, multiple sclerosis, motor neuron disease, epilepsy. NOTE: a person could be diagnosed with schizophrenia (i.e. they have a primary psychiatric psychotic disorder) but they also have a neurological disorder. |
| **Past neuro disorders affecting**  **the brain (specify)** | Free text to state what the past neurological disorders are |
| **All medications/drugs within 1**  **week before EEG** | Provide a list. If not stated, put 'NS' |

| **Number of subjects with**  **alcohol use within 1wk** | - |
| --- | --- |
| **Number of subjects**  **recreational drug use within 1wk** | - |
| **Number of subjects with**  **benzodiazepine use within 1wk** | - |
| **Number of subjects antipsychotics within 1wk**  **(including clozapine)** | - |
| **Number of subjects with**  **clozapine use within 1wk** | - |
| **Number of subjects with**  **antidepressants within 1wk** | - |
| **Duration of psychotic illness**  **(prior to EEG) (mean)** | Report in years |
| **Duration of psychotic illness**  **(prior to EEG) (SD)** | Report in years |
| **Diagnostic criteria used** | e.g. DSM-V or ICD-10 |
| **Type of EEG recording** | What type of EEG was used? E.g. scalp, intracranial. |
| **EEG results by diagnostic**  **group (image)** | - |
| **EEG results by diagnostic**  **group (text)** | - |
| **Number of subjects with**  **psychiatric psychosis** | - |
| **Psychiatric diagnosis (free text)** | Provide a breakdown of the final diagnoses with numbers. |
| **Number of subjects with organic**  **(secondary/neurological)**  **psychosis** | Provide a breakdown of the final diagnoses with numbers. |
| **Organic diagnosis (free text)** | - |
| **Extraction issues** | - |
| **Anything interesting?** | - |

**Supplementary Table 3:** Diagnostic odds ratios and likelihood ratios

| Study | Diagnostic Odds Ratio (95% CI) | Positive Likelihood ration (95% CI) | Negative Likelihood ration (95% CI) |
| --- | --- | --- | --- |
| Hinotsu et al. (2022) | 16.71 (3·90-71·56) | 4·02 (2·55-6·34) | 0·24 (0·08-0·74) |
| Endres et al. (2020) | 2·16 (0·04-109·30) | 1·58 (0·22-11·26) | 0·73 (0·10-5·20) |
| Restrepo-Martinez et al. (2020) | 6·23 (0·10-295·86) | 1·69 (0·24-12·06) | 0·31 (0·04-2·37) |
| Kim et al. (2019) | 0·45 (0·01-23·34) | 0·72 (0·10-5·17) | 1·62 (0·22-11·94) |
| Kroc et al. (2018) | 2·41 (0·05-127·68) | 1·70 (0·23-12·72) | 0·71 (0·10-5·07) |
| Endres et al. (2016) | 13·4 (0·26-695·87) | 7·20 (0·96-53·81) | 0·54 (0·08-3·82) |
| Chaychi et al. (2015) | 6·65 (0·12-357·76) | 3·82 (0·49-29·93) | 0·58 (0·08-4·09) |
| Okruszek et al. (2014) | 1·28 (0·03-64·99) | 1·14 (0·16-8·13) | 0·89 (0·13-6·34) |
| Raybould et al. (2012) | 4·07 (0·08-207·95) | 2·54 (0·35-18·31) | 0·62 (0·09-4·43) |
| Degner et al. (2011) | 1·42 (0·03-78·12) | 1·21 (0·16-9·12) | 0·85 (0·12-6·23) |
| Gschwandtner et al. (2009) | 3·27 (0·06-179·13) | 2·13 (0·27-16·70) | 0·65 (0·09-4·68) |
| Wichniak et al. (2006) | 1·03 (0·02-52·48) | 1·01 (0·14-7·25) | 0·98 (0·14-7·05) |
| Röttig et al. (2005) | 1·53 (0·03-78·24) | 1·26 (0·18-9·08) | 0·83 (0·12 – 5·91) |
| Pogarell et al. (2004) | 1·64 (0·03-84·44) | 1·32 (0·18-9·53) | 0·91 (0·11-5·76) |
| Amann et al. (2003) | 3·21 (0·06-166·92) | 2·10 (0·29-15·50) | 0·6 (0·09-4·67) |
| Schmitz et al. (1999) | 51·00 (0·41-6300·61) | 1·96 (0·28-13·94) | 0·04 (0·00-1·12) |
| Inui et al. (1998) | 7·63 (0·14-407·54) | 4·32 (0·56-33·50) | 0·57 (0·07-4·02) |
| Freudenreich et al. (1997) | 0·88 (0·02-46·15) | 0·94 (0·13-6·79) | 1·07 (0·15-7·78) |
| Neufeld et al. (1996) | 2·82 (0·05-159·96) | 1·48 (0·21-10·65) | 0·52 (0·07-4·22) |
| Kanemoto et al. (1996) | 10·87 (0·20-587·77) | 1·83 (0·26-13·02) | 0·17 (0·02-1·34) |
| Olesen et al. (1995) | 0·79 (0·02 – 41·20) | 0·90 (0·13-6·44) | 1·13 (0·16-8·20) |
| Haring et al. (1994) | 0·94 (0·02-46·15) | 0·94 (0·13-6·79) | 1·07 (0·15-7·78) |
| Green et al. (1992) | 0·43 (0·01-23·69) | 0·71 (0·10-5·16) | 1·67 (0·21-13·94) |
| Sandyk et al. (1992) | 4·05 (0·08-216·12) | 2·52 (0·33-19·28) | 0·62 (0·09-4·45) |
| Nagakubo et al. (1991) | 15·24 (0·28-816·07) | 8·12 (1·03-64·03) | 0·53 (0·08-3·78) |
| Koufen et al. (1987) | 201·00 (1·65-24543·34) | 1·99 (0·28-14·19) | 0·01 (0·00-0·29) |
| Kendler et al. (1982) | 0·55 (0·01-28·29) | 0·79 (0·11-5·53)) | 1·41 (0·20-10·15) |
| Guasp et al. (2021) | 34·71 (5·28-228·23) | 15·75 (4·03-61·49) | 0·45 (0·21-1·00) |
| Kikuchi et al. (2014) | 53·00 (0·43-6543·83) | 27·00 (0·93-788·06) | 0·51 (0·07-3·62) |
| Shrivastava et al. (2014) | 0·51 (0·01-26·51) | 0·76 (0·11-5·41) | 1·47 (0·20-10·70) |
| Price et al· (2002) | 1·45 (0·03-55·14) | 1·23 (0·17-9·03) | 0·84 (0·12-6·10) |
| Sandyk et al. (1993) | 3·71 (0·07-191·36) | 2·35 (0·32-17·21) | 0·64 (0·09-4·52) |
| Psatta et al. (1991) | 3·67 (0·06-211·75) | 1·57 (0·22-11·30) | 0·43 (0·05-3·59) |
| Wong et al. (1997) | 2·30 (0·04-123·39) | 1·65 (0·22-12·44) | 0·72 (0·10-5·15) |
| Welch et al. (1994) | 0·25 (0·00-13·56) | 0·62 (0·09-4·45) | 2·54 (0·32-20·27) |
| Small et al. (1984) | 1·53 (0·70-3·33) | 1·26 (0·85-1·88) | 0·83 (0·56-1·22) |
| Jefsen et al. (2023) | 3·06 (0·06-159·88) | 2·03 (0·27-15·02) | 0·66 (0·09-4·73) |
| Tsutsui et al. (2012) | 9·80 (1·59-60·44) | 4·67 (1·74–12·53) | 0·48 (0·18–1·23) |

**Supplementary Table 4:** EEG report proforma used for both primary and secondary cases

| Question | Response |
| --- | --- |
| EEG normal | Number of EEGs considered normal |
| EEG abnormal | Number of EEGs considered abnormal |
| Features of encephalopathy | Number of EEGs with features of encephalopathy (e.g. background slowing) present |
| If features of encephalopathy, background rhythm predominantly in theta range (4 - <8 Hz) | Number of EEGs with background rhythm predominantly in theta range |
| If features of encephalopathy, background rhythm predominantly in delta range (0.1 - <4 Hz) | Number of EEGs with background rhythm predominantly in delta range |
| If features of encephalopathy, background rhythm showing mixed theta-delta activity | Number of EEGs with background rhythm showing mixed theta-delta activity |
| Background asymmetry between hemispheres | Number of EEGs with background asymmetry between hemispheres |
| Non-epileptiform focal abnormality | Number of EEGs with non-epileptiform focal abnormality |
| Rhythmic theta or delta activity | Number of EEGs with rhythmic theta or delta activity |
| Features of limbic encephalitis | Number of EEGs with features of limbic encephalitis (e.g. extreme delta brush) present |
| Any epileptiform discharges | Number of EEGs with epileptiform discharges present, including active seizure and interictal epileptiform discharges |
| If any epileptiform discharges, number focal | Number of EEGs with focal epileptiform discharges |
| If any epileptiform discharges, number generalised | Number of EEGs with generalised epileptiform discharges |
| Electrographic seizures | Number of EEGs with electrographic seizures |
| Non-convulsive status epilepticus | Number of EEGs with non-convulsive status epilepticus |
| Convulsive seizures | Number of EEGs with convulsive seizures |
| If convulsive seizures, convulsive status epilepticus | Number of EEGs with convulsive status epilepticus |
| Any periodic activity | Number of EEGs with periodic activity |
| If any periodic activity, number lateralised | Number of EEGs with lateralised periodic activity |
| If any periodic activity, number generalised | Number of EEGs with generalised periodic activity |

**Supplementary Table 5:** Adapted QUADAS-2 tool

| **Signalling questions** | **Recording options** |
| --- | --- |
| Author | Free text |
| Describe methods of patient selection | Free text |
| Was a consecutive or random sample of patients enrolled? | Yes/ No/ Unclear |
| Was a case-control design avoided? | Yes/ No/ Unclear |
| Did the study avoid inappropriate exclusions? | Yes/ No/ Unclear |
| Could the selection of patients have introduced bias? | High risk of bias/ Low risk of bias/  Unclear |
| Describe included patients (prior testing, presentation,  intended use of index test and setting) | Free text |
| Are there concerns that the included patients do not match the  review questions? | High concerns/ Low concerns/  Unclear |
| Describe the index test and how it was conducted and  interpreted | Free text |
| Were the index test results interpreted without knowledge of  the results of the reference standard? | Yes/ No/ Unclear |
| If a threshold was used, was it prespecified? | Yes/ No/ Unclear |
| Could the conduct or interpretation of the index test have  introduced bias? | High risk of bias/ Low risk of bias/  Unclear |
| Are there concerns that the index test, its conduct, or  interpretation differ from the review question? | High concerns/ Low concerns/  Unclear |
| Describe the reference standard and how it was conducted  and interpreted | Free text |
| Confidence in the clinical diagnosis (reference standard): were  patients followed up for at least 6 months? | Yes/ No/ Unclear |
| Were the reference standard results interpreted without  knowledge of the results of the index test? | Yes/ No/ Unclear |
| Could the reference standard, its conduct, or its interpretation  have introduced bias? | High risk of bias/ Low risk of bias/  Unclear |
| Are there concerns that the target condition as defined by the  reference standard does not match the review question? | High concerns/ Low concerns/  Unclear |
| Describe any patients who did not receive the index test(s) and/or reference standard or who were excluded from the 2x2  table | Free text |
| Describe the time interval and any interventions between index  test(s) and reference standard | Free text |
| Was there an appropriate interval between index test and  reference standard | Yes/ No/ Unclear |
| Did all patients receive a reference standard? | Yes/ No/ Unclear |
| Did patients receive the same reference standard? | Yes/ No/ Unclear |
| Were all patients included in the analysis? | Yes/ No/ Unclear |
| Could the patient flow have introduced bias? | High risk of bias/ Low risk of bias/  Unclear |

**Supplementary Table 6:** Details of studies excluded at full text screening

| **Name of Study** | **First Author** | **Year of Publication** | **Journal/Book Title** | **Reason for exclusion** |
| --- | --- | --- | --- | --- |
| Clinical features, treatments, and outcomes of patients with anti-N-methyl-D-aspartate encephalitis-a single-center, retrospective analysis in China. | Xu et al | 2021 | Frontiers in Bioscience | No psychotic diagnosis |
| Abnormal phase discontinuity of alpha- and theta-frequency oscillations in schizophrenia. | Koshiyama et al | 2021 | Schizophrenia Research | Quantitative EEG |
| Clinical-neurophysiological correlations in patients with depressive-delusional conditions | Iznak et al | 2021 | Zhurnal Nevrologii | Quantitative EEG |
| Distinct alterations in resting-state electroencephalogram during eyes closed and eyes open and between morning and evening are present in first-episode psychosis patients. | Zhang et al | 2021 | Schizophrenia Research | Quantitative EEG |
| Alterations of Resting EEG in Hallucinating and Nonhallucinating Schizophrenia Patients. | Arora et al | 2021 | Clinical EEG & Neuroscience | Quantitative EEG |
| Abnormalities of resting-state EEG in patients with prodromal and overt dementia with Lewy bodies: Relation to clinical symptoms. | Pascarelli et al | 2020 | Clinical Neurophysiology | Quantitative EEG |
| Myoclonic epilepsy, parkinsonism, schizophrenia and left-handedness as common neuropsychiatric features in 22q11.2 deletion syndrome. | Fanella et al | 2020 | Journal of Medical Genetics | No psychotic diagnosis |
| Abnormalities in hubs location and nodes centrality predict cognitive slowing and increased performance variability in first-episode schizophrenia patients. | Krukow et al | 2019 | Scientific Reports | Quantitative EEG |
| Topographic deficits in sleep spindle density and duration point to frontal thalamo-cortical dysfunctions in first-episode psychosis. | Kaskie et al | 2019 | Journal of Psychiatric Research | EEG only during sleep |
| Overview of the clinical implementation of a study exploring social withdrawal in patients with schizophrenia and Alzheimer's disease. | Bilderbeck et al | 2019 | Neuroscience & Biobehavioral Reviews | No EEG report |
| Electroencephalogram Modifications Associated With Atypical Strict Antipsychotic Monotherapies. | Dias-Alves et al | 2018 | Journal of Clinical Psychopharmacology | No psychotic diagnosis |
| Investigation of the Video-EEG Findings and Clinical Data in Patients Diagnosed With Epilepsy and Psychosis. | Mehdikhanova et al | 2018 | Neurologist | n < 20 |
| A Preliminary Study of Central Nervous System Arousal and Sleep Quality in Bipolar Disorder. | Cardinale et al | 2018 | Psychopathology | No psychotic diagnosis |
| Nineteen and Up study (19Up): understanding pathways to mental health disorders in young Australian twins. | Couvy-Duchesne et al | 2018 | BMJ Open | No EEG report |
| Abnormal functional connectivity of high-frequency rhythms in drug-naive schizophrenia. | Takahashi et al | 2018 | Clinical Neurophysiology | Quantitative EEG |
| Local and Widely Distributed EEG Activity in Schizophrenia With Prevalence of Negative Symptoms. | Grin-Yatsenko et al | 2017 | Clinical EEG & Neuroscience | Quantitative EEG |
| Deconstructing Bipolar Disorder and Schizophrenia: A cross-diagnostic cluster analysis of cognitive phenotypes. | Lee et al | 2017 | Journal of Affective Disorders | Quantitative EEG |
| Post-steroid neuropsychiatric manifestations are significantly more frequent in SLE compared with other systemic autoimmune diseases and predict better prognosis compared with de novo neuropsychiatric SLE. | Shimizu et al | 2016 | Autoimmunity Reviews | No psychotic diagnosis |
| The course of negative symptoms over the first five years of treatment: Data from an early intervention program for psychosis. | Norman et al | 2015 | Schizophrenia Research | No psychotic diagnosis |
| Electroencephalographic theta activity and cognition in schizophrenia: preliminary results. | Wichniak et al | 2015 | World Journal of Biological Psychiatry | No EEG report |
| Electroencephalographic abnormalities and 5-year outcome in first-episode psychosis. | Manchanda et al | 2014 | Canadian Journal of Psychiatry - Revue Canadienne de Psychiatrie | Duplicate |
| A study on validity of cortical alpha connectivity for schizophrenia. | Peng et al | 2013 | Annual International Conference Of The IEEE Engineering In Medicine And Biology Society | Quantitative EEG |
| Diagnosis and treatments of Hashimoto's encephalopathy | Yoneda | 2012 | Rinsho Shinkeigaku - Clinical Neurology | No EEG report |
| The clinical presentation and imaging manifestation of psychosis and dementia in general paresis: a retrospective study of 116 cases. | Zheng et al | 2011 | Journal of Neuropsychiatry & Clinical Neurosciences | No psychotic diagnosis |
| EEG does not predict response of manic patients to atypical antipsychotics. | Reeves et al | 2011 | Clinical EEG & Neuroscience | Not original research |
| Ziprasidone vs olanzapine in recent-onset schizophrenia and schizoaffective disorder: results of an 8-week double-blind randomized controlled trial. | Grootens et al | 2011 | Schizophrenia Bulletin | No EEG report |
| Thalamic dysfunction in schizophrenia suggested by whole-night deficits in slow and fast spindles. | Ferrarelli et al | 2010 | American Journal of Psychiatry | EEG only during sleep |
| Slow wave sleep deficits as a trait marker in patients with schizophrenia. | Sarkar et al | 2010 | Schizophrenia Research | EEG only during sleep |
| Clinical and EEG approach to the diagnosis of epilepsy in a population of old people with delusion | Koskas et al | 2010 | Revue Neurologique | Not in English |
| EEG abnormalities and 3-year outcome in first episode psychosis. | Manchanda et al | 2008 | Acta Psychiatrica Scandinavica | Duplicate |
| The significance of family history in first-episode schizophrenia spectrum disorder. | Norman et al | 2007 | Journal of Nervous & Mental Disease | Duplicate |
| Corpora amylacea in mesial temporal lobe epilepsy: clinico-pathological correlations. | Radhakrishnan et al | 2007 | Epilepsy Research | No EEG report |
| Psychoses in patients with Parkinson's disease; their frequency, phenomenology, and clinical correlates | Kashihara et al | 2005 | Rinsho Shinkeigaku - Clinical Neurology | Untraceable full text |
| EEG abnormalities and two year outcome in first episode psychosis. | Manchanda et al | 2005 | Acta Psychiatrica Scandinavica | No EEG report |
| Decreased nonlinear complexity and chaos during sleep in first episode schizophrenia: a preliminary report. | Keshavan et al | 2004 | Schizophrenia Research | n < 20 |
| EEG abnormalities and outcome in first-episode psychosis. | Manchanda et al | 2003 | Canadian Journal of Psychiatry - Revue Canadienne de Psychiatrie | Duplicate |
| EEG changes with antipsychotic drugs. | Fink | 2002 | American Journal of Psychiatry | Not original research |
| A comparison of risk factors for habitual violence in pre-trial subjects. | Kaliski | 2002 | Acta Psychiatrica Scandinavica | No psychotic diagnosis |
| EEG abnormalities during treatment with typical and atypical antipsychotics. | Centorrino et al | 2002 | American Journal of Psychiatry | No psychotic diagnosis |
| Abnormal EEG patterns in treatment-resistant schizophrenic patients. | Ramos et al | 2001 | International Journal of Neuroscience | n < 20 |
| Reexamination of interictal psychoses based on DSM IV psychosis classification and international epilepsy classification. | Kanemoto et al | 2001 | Epilepsia | No EEG report |
| Clinical and biological concomitants of resting state EEG power abnormalities in schizophrenia. | Sponheim et al | 2000 | Biological Psychiatry | Quantitative EEG |
| Electroencephalogram alterations during treatment with olanzapine. | Pillmann et al | 2000 | Psychopharmacology | No psychotic diagnosis |
| An electroencephalographic investigation of late-onset schizophrenia. | Sachdev et al | 1999 | International Psychogeriatrics | Quantitative EEG |
| The electroencephalogram in psychiatric patients | Weiser et al | 1998 | Harefuah | Not in English |
| A longitudinal study of EEG sleep in schizophrenia. | Keshavan et al | 1996 | Psychiatry Research | EEG only during sleep |
| EEG abnormalities before clozapine therapy predict a good clinical response to clozapine. | Pillay et al | 1996 | Annals of Clinical Psychiatry | Untraceable full text |
| Clinical electroencephalograms in patients with catatonic disorders. | Carroll and Boutros | 1995 | Clinical Electroencephalography | n < 20 |
| Resting EEG in first-episode and chronic schizophrenia. | Sponheim et al | 1994 | Psychophysiology | Quantitative EEG |
| Correlation of third ventricular enlargement and EEG slow wave activity in schizophrenic patients. | Takeuchi et al | 1994 | Psychiatry Research | Quantitative EEG |
| Resting EEG in first-episode schizophrenia patients, bipolar psychosis patients, and their first-degree relatives. | Clementz et al | 1994 | Psychophysiology | Quantitative EEG |
| Epileptic seizures in patients with acute catatonic syndrome. | Primavera et al | 1994 | Journal of Neurology, Neurosurgery & Psychiatry | No EEG report |
| CSF levels of diazepam-binding inhibitor correlate with REM latency in schizophrenia, a pilot study. | van Kammen et al | 1994 | European Archives of Psychiatry & Clinical Neuroscience | EEG only during sleep |
| EEG alterations and seizures during treatment with clozapine. A retrospective study of 283 patients. | Gunther et al | 1993 | Pharmacopsychiatry | No psychotic diagnosis |
| A clinical study on intellectual impairment in parkinsonian patients during long-term treatment. | Horiguchi et al | 1991 | Japanese Journal of Psychiatry & Neurology | No psychotic diagnosis |
| Clozapine in the treatment of 121 out-patients. | Leppig et al | 1989 | Psychopharmacology | No psychotic diagnosis |
| Comparison of untreated and treated schizophrenic patients, normals, and neuroleptic-treated normals: "hypofrontality" and different EEG spectra before and during voluntary movement. |  | 1989 | Psychiatry Research | Quantitative EEG |
| Bipolar affective disorder. II. EEG, neuropsychological, and clinical correlates of CT abnormality. | Dewan et al | 1988 | Acta Psychiatrica Scandinavica | No psychotic diagnosis |
| Clinical and EEG studies of zotepine, a thiepine neuroleptic, on schizophrenic patients. | Higashi et al | 1987 | Pharmacopsychiatry | n < 20 |
| EEG abnormalities in bipolar affective disorder. | Cook et al | 1986 | Journal of Affective Disorders | No psychotic diagnosis |
| Diminished order in the EEG of schizophrenic patients. | Diekmann et al | 1985 | Naturwissenschaften | No EEG report |
| Schizoaffective psychoses in Germany and Japan--a transcultural psychiatric study | Omata | 1985 | Fortschritte der Neurologie-Psychiatrie | Not in English |
| Exogenous psychoses in Parkinson syndrome. Frequency and causal conditions | Schneider et al | 1984 | Fortschritte der Neurologie-Psychiatrie. | Not in English |
| Carbamazepine in chronic patients with EEG abnormalities. | Kuehnle et al | 1984 | Journal of Clinical Psychiatry | n < 20 |
| Bilateral electrodermal habituation-dishabituation and resting EEG in remitted schizophrenics. | Iacono | 1982 | Journal of Nervous & Mental Disease | Quantitative EEG |
| Early- and late-onset bipolar illness. | Taylor and Abrams | 1981 | Archives of General Psychiatry | No EEG report |
| Delusional misidentification syndromes and cerebral 'dysrhythmia'. | Christodoulou and Malliara-Loulakaki | 1981 | Psychiatria Clinica. | No EEG report |
| An objective study of relationships and discontinuities between paranoid schizophrenia and Kretschmer's syndrome of sensitive delusions of reference. | Douglass and Hays P | 1980 | Acta Psychiatrica Scandinavica | No EEG report |
| Neuropsychiatric lupus. | Abel Tet al | 1980 | Journal of Rheumatology | No psychotic diagnosis |
| Using multivariate endophenotypes to identify psychophysiological mechanisms associated with polygenic scores for substance use, schizophrenia, and education attainment. | Harper et al | 2022 | Psychological Medicine | n < 20 |
| Correlation between resting theta power and cognitive performance in patients with schizophrenia. | Cao et al | 2022 | Frontiers in Human Neuroscience | Quantitative EEG |
| Delirious mania as a frequent and recognizable neuropsychiatric syndrome in patients with anti-NMDAR encephalitis | Restrepo-Martinez et al | 2021 | General Hospital Psychiatry | Duplicate |
| Clinical-neurophysiological correlations in patients with depression-delusional conditions. | Iznak et al | 2022 | Neuroscience and Behavioral Physiology | Quantitative EEG |
| Abnormal phase discontinuity of alpha- and theta-frequency oscillations in schizophrenia. | Koshiyama et al | 2021 | Schizophrenia Research | Quantitative EEG |
| Cognitive impairment and diminished neural responses constitute a biomarker signature of negative symptoms in psychosis. | Hudgens-Haneye et al | 2020 | Schizophrenia Bulletin | Quantitative EEG |
| Delirious mania as a frequent and recognizable neuropsychiatric syndrome in patients with anti-NMDAR encephalitis. | Restrepo-Martinez et al | 2020 | General Hospital Psychiatry | Duplicate |
| Common and distinct global functional connectivity density alterations in patients with bipolar disorder with and without auditory verbal hallucination during major depressive episodes. | Qiu et al | 2020 | Brain Imaging and Behavior | No EEG report |
| Cariprazine safety in adolescents and the elderly: Analyses of clinical study data. | Szatmari et al | 2020 | Frontiers in Psychiatry | No EEG report |
| Treatment emergent affective switch with intermittent theta burst stimulation over right temporo-parietal junction: A case report. | Garg et al | 2020 | Brain Stimulation | n < 20 |
| Autoimmune encephalitis with psychosis: Warning signs, step-by-step diagnostics and treatment. | Steiner et al | 2020 | The World Journal of Biological Psychiatry | Not original research (e.g. review articles) |
| Association between electroencephalogram changes and plasma clozapine levels in clozapine-treated patients. | Kim et al | 2019 | International Clinical Psychopharmacology | Duplicate |
| Topographic deficits in sleep spindle density and duration point to frontal thalamo-cortical dysfunctions in first-episode psychosis. | Kaskie et al | 2019 | Journal of Psychiatric Research | EEG only during sleep |
| Sertindole: EEG analysis, tolerability, and clinical efficacy. | Kroc et al | 2018 | Pharmacopsychiatry | Duplicate |
| Deconstructing Bipolar Disorder and Schizophrenia: A cross-diagnostic cluster analysis of cognitive phenotypes. | Lee et al | 2017 | Journal of Affective Disorders | Quantitative EEG |
| Immunological findings in psychotic syndromes: A tertiary care hospital's CSF sample of 180 patients. | Endres et al | 2015 | Frontiers in Human Neuroscience | No psychotic diagnosis |
| The course of negative symptoms over the first five years of treatment: Data from an early intervention program for psychosis. | Norman et al | 2015 | Schizophrenia Research | Duplicate |
| Electroencephalographic theta activity and cognition in schizophrenia: Preliminary results. | Wichniak et al | 2015 | The World Journal of Biological Psychiatry | Quantitative EEG |
| Electroencephalographic characteristics of Iranian schizophrenia patients. | Chaychi et al | 2015 | Acta Neurologica Belgica | Duplicate |
| Electroencephalographic abnormalities and 5-year outcome in first-episode psychosis. | Manchanda et al | 2014 | The Canadian Journal of Psychiatry / La Revue canadienne de psychiatrie | Duplicate |
| EEG screening for temporal lobe epilepsy in patients with acute psychosis. | Raybould et al | 2012 | The Journal of Neuropsychiatry and Clinical Neurosciences | Duplicate |
| EEG alterations during treatment with olanzapine. | Degner et al | 2011 | European Archives of Psychiatry and Clinical Neuroscience | Duplicate |
| The clinical presentation and imaging manifestation of psychosis and dementia in general paresis: A retrospective study of 116 cases. | Zheng et al | 2011 | The Journal of Neuropsychiatry and Clinical Neurosciences | No psychotic diagnosis |
| Ziprasidone vs olanzapine in recent-onset schizophrenia and schizoaffective disorder: Results of an 8-week double-blind randomized controlled trial. | Grootens et al | 2011 | Schizophrenia Bulletin | No EEG report |
| Biopsychosocial and forensic clinical correlates of schizophrenia and homicide. | Schug, Robert | 2010 | Dissertation Abstracts International: Section B: The Sciences and Engineering | Not peer-reviewed |
| Slow wave sleep deficits as a trait marker in patients with schizophrenia. | Sarkar et al | 2010 | Schizophrenia Research | Quantitative EEG |
| Thalamic dysfunction in schizophrenia suggested by whole-night deficits in slow and fast spindles. | Ferrarelli et al | 2010 | The American Journal of Psychiatry | Duplicate |
| Clinical and EEG approach to the diagnosis of epilepsy in a population of old people with delusion. | Koskas et al | 2010 | Revue Neurologique | Duplicate |
| EEG: A helpful tool in the prediction of psychosis. | Gschwandtner et al | 2009 | European Archives of Psychiatry and Clinical Neuroscience | Duplicate |
| EEG abnormalities and 3-year outcome in first episode psychosis. | Manchanda et al | 2008 | Acta Psychiatrica Scandinavica | Duplicate |
| The significance of family history in first-episode schizophrenia spectrum disorder. | Norman et al | 2007 | Journal of Nervous and Mental Disease | Duplicate |
| Is There Evidence in the EEG for Increased Epileptiform Activity in ICD-10 Acute and Transient Psychotic Disorder? | Rottig et al | 2005 | Psychopathology | Duplicate |
| EEG abnormalities and two year outcome in first episode psychosis. | Manchanda et al | 2005 | Acta Psychiatrica Scandinavica | Duplicate |
| Olanzapine (Zyprexa) Treatment in Patients Pre-treated with other Antipsychotics: Pharmacovigilance Data from a Large Drug Utilization Observation (DUO) Study in Germany. | Czekalla et al | 2005 | German Journal of Psychiatry | No EEG report |
| Influence of family, perinatal and traumatic background, as well as eletroencephalographic abnormalities in the clinical characteristics of schizophrenic patients.. | Leon and Gonzales | 2004 | Psiquiatria | Untraceable full text |
| EEG Abnormalities Under Treatment with Atypical Antipsychotics: Effects of Olanzapine and Amisulpride as Compared to Haloperidol. | Pogarell et al | 2004 | Pharmacopsychiatry | Duplicate |
| Decreased nonlinear complexity and chaos during sleep in first episode schizophrenia: a preliminary report. | Keshavan et al | 2004 | Schizophrenia Research | Duplicate |
| EEG abnormalities associated with antipsychotics: A comparison of quetiapine, olanzapine, haloperidol and healthy subjects. | Amann et al | 2003 | Human Psychopharmacology: Clinical and Experimental | Duplicate |
| Studies of individuals with schizophrenia never treated with antipsychotic medications: A review. | Torrey, E. Fuller | 2002 | Schizophrenia Research | Not original research |
| EEG abnormalities before clozapine therapy predict a good clinical response to clozapine. | Pillay et al | 1996 | Annals of Clinical Psychiatry | Duplicate |
| Interpretation of electroencephalograms in psychotic subjects. | Faber et al | 1987 | Cesko-Slovenska Psychiatrie | Not in English |
| Reexamination of interictal psychoses based on DSM IV psychosis classification and international epilepsy classification. | Kanemoto et al | 2001 | Epilepsia | Not original research |
| Abnormal EEG patterns in treatment-resistant schizophrenic patients. | Ramos et al | 2001 | International Journal of Neuroscience | Duplicate |
| The clinical applications of Mini-Mental State Examination in geropsychiatric inpatients. | Yang et al | 2000 | International Journal of Psychiatry in Medicine | No EEG report |
| Clinical and biological concomitants of resting state EEG power abnormalities in schizophrenia. | Sponheim et al | 2000 | Biological Psychiatry | Quantitative EEG |
| Electroencephalogram alterations during treatment with olanzapine. . | Pillmann et al | 2000 | Psychopharmacology | Duplicate |
| An electroencephalographic investigation of late-onset schizophrenia. | Sachdev et al | 1999 | International Psychogeriatrics | Duplicate |
| Electroencephalographic findings in patients with DSM-IV mood disorder, schizophrenia, and other psychotic disorders. | Inui et al | 1998 | Biological Psychiatry | Duplicate |
| Intra- and interhemispheric electroencephalogram coherence in siblings discordant for schizophrenia and healthy volunteers. | Mann et al | 1997 | Biological Psychiatry | Quantitative EEG |
| Clozapine-induced electroencephalogram changes as a function of clozapine serum levels. | Freudenreich et al | 1997 | Biological Psychiatry | Duplicate |
| Electroencephalographic findings with low-dose clozapine treatment in psychotic Parkinsonian patients. | Neufeld et al | 1996 | Clinical Neuropharmacology | Duplicate |
| A longitudinal study of EEG sleep in schizophrenia. | Keshavan et al | 1995 | Psychiatry Research | EEG only during sleep |
| Clozapine serum levels and side effects during steady state treatment of schizophrenic patients: A cross-sectional study. | Olesen et al | 1995 | Psychopharmacology | Duplicate |
| Epileptic seizures in patients with acute catatonic syndrome. | Primavera et al | 1994 | Journal of Neurology, Neurosurgery & Psychiatry | No EEG report |
| Resting EEG in first-episode schizophrenia patients, bipolar psychosis patients, and their first-degree relatives. | Clementz et al | 1994 | Psychophysiology | Quantitative EEG |
| Clozapine-induced seizures and EEG changes. | Welch et al | 1994 | The Journal of Neuropsychiatry and Clinical Neurosciences | Duplicate |
| Correlation of third ventricular enlargement and EEG slow wave activity in schizophrenic patients. | Takeuchi et al | 1994 | Psychiatry Research: Neuroimaging | Quantitative EEG |
| EEG alterations in patients treated with clozapine in relation to plasma levels. | Haring et al | 1994 | Psychopharmacology | Duplicate |
| Hyperreligiosity in psychotic disorders. | Brewerton, Timothy | 1994 | Journal of Nervous and Mental Disease | Study only included patients with abnormal EEGs at the start |
| Resting EEG in first-episode and chronic schizophrenia. | Sponheim et al | 1994 | Psychophysiology | Quantitative EEG |
| EEG alterations and seizures during treatment with clozapine: A retrospective study of 283 patients. | Gunther et al | 1993 | Pharmacopsychiatry | Duplicate |
| The delusional misidentification syndromes in patients with and without evidence of organic cerebral disorder: A structured review of case reports. | Fleminger and Burns | 1993 | Biological Psychiatry | No EEG report |
| Clozapine for schizophrenia. | Blanz and Schmidt | 1993 | Journal of the American Academy of Child & Adolescent Psychiatry | Not peer-reviewed |
| A comparative study of EEG of normal subjects and schizophrenic patients. | Yan et al | 1992 | Psychological Science | Not in English |
| Schizophrenia with childhood onset: A phenomenological study of 38 cases. | Green et al | 1992 | Journal of the American Academy of Child & Adolescent Psychiatry | Duplicate |
| Adolescent developmental change in topography of EEG amplitude. | Buchsbaum et al | 1992 | Schizophrenia Research | No psychotic diagnosis |
| Abnormal EEG and calcification of the pineal gland in schizophrenia. | Sandyk and Kay | 1992 | International Journal of Neuroscience | Duplicate |
| Lithium-neuroleptic interactions: Electroencephalographic studies. | Wolf et al | 1991 | Research Communications in Psychology, Psychiatry & Behavior | No psychotic diagnosis |
| New-onset psychosis in HIV-infected patients. | Harris et al | 1991 | The Journal of Clinical Psychiatry | Not original research |
| Carbamazepine compared with lithium in the treatment of mania. | Small et al | 1991 | Archives of General Psychiatry | No psychotic diagnosis |
| Drug treatment of schizophrenia: Current concepts. | Naber and Hippius | 1991 | Pharmacopsychiatry | Not original research |
| The patients of a special psychogeriatric ward: Psychosocial situation, clinical disorder and results of treatment. | Salokangas et al | 1990 | Psychiatria Fennica | Untraceable full text |
| Mechanisms of action of ECT: Schizophrenia and schizoaffective disorder. | Milstein et al | 1990 | Biological Psychiatry | No EEG report |
| Clozapine in the treatment of 121 out-patients. | Leppig et al | 1989 | Psychopharmacology | Duplicate |
| Comparison of untreated and treated schizophrenic patients, normals, and neuroleptic-treated normals: "Hypofrontality" and different EEG spectra before and during voluntary movement. | Westphal et al | 1989 | Psychiatry Research | Quantitative EEG |
| Schizophrenic syndromes in epilepsies. | Diehl, Lothar | 1989 | Psychopathology | Not original research |
| EEG correlates of clinical heterogeneity of schizophrenia. | Kemali et al | 1988 | The EEG of mental activities | Quantitative EEG |
| Psychoses of Down's syndrome children and normal children. [Italian]. | Cocchi, Renato | 1988 | Italian Journal of Intellective Impairment | Not in English |
| EEG and autonomic characteristics not correlated with normal and DAF speech characteristics. | Irmis et al | 1988 | Activitas Nervosa Superior | Untraceable full text |
| Systematic EEG follow-up study of traumatic psychosis. | Koufen and Hagel | 1987 | European Archives of Psychiatry & Neurological Sciences | Duplicate |
| Clinical and ethiopathogenetical aspects of the epileptic psychosis without clouding of conscience. | Dorr Zegers and Rauh | 1984 | Actas Luso-Espanolas de Neurologia, Psiquiatria y Ciencias Afines | Not in English |
| EEG abnormalities in bipolar affective disorder. | Cook et al | 1986 | Journal of Affective Disorders | Duplicate |
| Schizophrenia complicated by a craniocerebral trauma and other diseases of exogenous-organic nature. | Golodets and Fedorenko | 1986 | Zhurnal Nevropatologii i Psikhiatrii | Not in English |
| Study of 60 inmates with epileptic psychoses in the Psychiatric Hospital of Havana. | Gonzalez et al | 1985 | Revista del Hospital Psiquiatrico de La Habana | Not in English |
| Psychopathological differences between clear consciousness epileptic psychosis and similar psychosis with EEG alterations but without epilepsy. | Parada et al | 1985 | Revista Chilena de Neuro-psiquiatria | Not in English |
| Schizoaffective psychoses in Germany and in Japan: A transcultural psychiatric study. | Omata, Waichiro | 1985 | Fortschritte der Neurologie, Psychiatrie. | Duplicate |
| Frequency and cause of psychotic symptomatology in Parkinson's disease. | Schneider et al | 1984 | Fortschritte der Neurologie, Psychiatrie. | Not original research (e.g. review articles) |
| A comparison of puerperal psychosis and the schizophreniform variant of manic-depression. | Hays and  Douglass | 1984 | Acta Psychiatrica Scandinavica | No EEG report |
| Evolution of 25 children with schizomorphic psychosis and its relationship to epilepsy and brain damage. | de la Barra Mac-Donald, Flora | 1983 | Revista Chilena de Neuro-psiquiatria | Not in English |
| Electroencephalographic studies of psychotic children and their families. | Antonovic and  Mitrovic | 1983 | Psihijatrija Danas | Not in English |
| Familial and sporadic schizophrenia: A symptomatic, prognostic, and EEG comparison. | Kendler and  Hays | 1982 | The American Journal of Psychiatry | Duplicate |
| Alzheimer's disease and senile dementia of Alzheimer type: A comparative study. | Sulkava, Raimo | 1982 | Acta Neurologica Scandinavica | No psychotic diagnosis |
| Bilateral electrodermal habituation-dishabituation and resting EEG in remitted schizophrenics. | Iacono et al | 1982 | Journal of Nervous and Mental Disease | Quantitative EEG |
| Delusional misidentification syndromes and cerebral "dysrhythmia." |  | 1981 | Psychiatria Clinica | No EEG report |
| Early- and late-onset bipolar illness. | Taylor and Abrams | 1981 | Archives of General Psychiatry | Duplicate |
| An objective study of relationships and discontinuities between paranoid schizophrenia and Kretschmer's syndrome of sensitive delusions of reference. | Douglass and Hays | 1980 | Acta Psychiatrica Scandinavica | Duplicate |
| Neural autoantibodies in cerebrospinal fluid and serum in clinical high risk for psychosis, first-episode psychosis, and healthy volunteers | Bien et al | 2021 | Frontiers in Psychiatry | Only included patients who had normal EEG |
| Neuropharmacological computational analysis of longitudinal electroencephalograms in clozapine-treated patients with schizophrenia using hierarchical dynamic causal modeling | Eo et al | 2023 | NeuroImage | Quantitative EEG |
| The EEG multiverse of schizophrenia | Gordillo et al | 2023 | Cerebral Cortex | Quantitative EEG |
| The validity of atypical psychosis diagnostic criteria to detect anti-NMDA receptor encephalitis with psychiatric symptoms | Hintosu et al | 2022 | Schizophrenia Research | Duplicate |
| Investigating neurophysiological markers of impaired cognition in schizophrenia | Hoy et al | 2021 | Schizophrenia Research | Quantitative EEG |
| Risk factors for clozapine-induced central nervous system abnormalities in Japanese patients with treatment-resistant schizophrenia | Kitagawa et al | 2021 | Asian Journal of Psychiatry | Only included patients with abnormal EEG |
| Correlation study of brain-derived neurotrophic factor, EEG gamma activity and cognitive function in first-episode schizophrenia | Li et al | 2023 | Brain Research | Quantitative EEG |
| Shared and Distinct Topographic Alterations of Alpha-Range Resting EEG Activity in Schizophrenia, Bipolar Disorder, and Depression | Xue et al | 2023 | Neuroscience bulletin | Quantitative EEG |
| Resting-State EEG Connectivity at High-Frequency Bands and Attentional Performance Dysfunction in Stabilized Schizophrenia Patients | Yeh et al | 2023 | Medicina (Kaunas, Lithuania) | Quantitative EEG |
| Sertindole: EEG Analysis, Tolerability, and Clinical Efficacy. | Kroc et al | 2018 | Pharmacopsychiatry | Duplicate |
| Daytime sleepiness and EEG abnormalities in patients treated with second generation antipsychotic agents | Okruszek et al | 2014 | Pharmacol Reports | Duplicate |
| Electroencephalogram slowing, sleepiness and treatment response in patients with schizophrenia | Wichniak et al | 2006 | Journal of Psychopharmacology | Duplicate |
| The empirical mode decomposition and Lempel-Ziv complexity. The new possibility in diagnosis of EEG in schizophrenic patients? | Latka et al | 2010 | Przeglad Lekarski | n < 20 |
| Increased Prevalence of Diverse N-Methyl- D -Aspartate Glutamate Receptor Antibodies in Patients with an Initial Diagnosis of Schizophrenia | Steiner et al | 2013 | JAMA Psychiatry | n < 20 |
| Comparisons between Psychiatric Symptoms of Patients with Anti-NMDAR Encephalitis and New-Onset Psychiatric Patients | Wang et al | 2017 | Neuropsychobiology | No EEG report |

**Supplementary Table 7:** Sensitivity analyses varying the continuity correction

| ***Continuity correction*** | **Sensitivity (95%CI)** | **Specificity (95%CI)** | **I²** |
| --- | --- | --- | --- |
| *0*·*5 (main analysis)* | 0·71 (0·61 – 0·80) | 0·67 (0·58 – 0·74) | 9·2% |
| *0*·*1* | 0·76 (0·64 – 0·85) | 0·68 (0·59 – 0·75) | 4·2% |
| *0*·*01* | 0·78 (0·65 – 0·87) | 0·68 (0·59 – 0·75) | 0·6% |
| *1 * 10^-4^* | 0·78 (0·65 – 0·87) | 0·68 (0·59 – 0·75) | 0·0% |
| *1 * 10^-8^* | 0·78 (0·65 – 0·87) | 0·68 (0·59 – 0·75) | 0·0% |

**Supplementary Table 8:** Subgroup analyses by study type

| ***Subgroup analysis*** | **No. of studies** | **No. of patients (patients with abnormality)** | **Sensitivity (95%CI)** | **Specificity (95%CI)** | **AUC** | **I²** |
| --- | --- | --- | --- | --- | --- | --- |
| *Main analysis* | 38 | 3784 (1409) | 0·71 (0·61-0·80) | 0·67 (0·58-0·74) | 0·67 | 9·2% |
| *Cohort studies only* | 6 | 915 (334) | 0·69 (0·44 - 0·87) | 0·76 (0·59 - 0·88) | 0·57 | 0·0% |
| *Case series only* | 32 | 2869 (1075) | 0·76 (0·68 - 0·83) | 0·65 (0·55 - 0·73) | 0·86 | 0·0% |

**Supplementary Figure 1:** Fagan’s nomogram


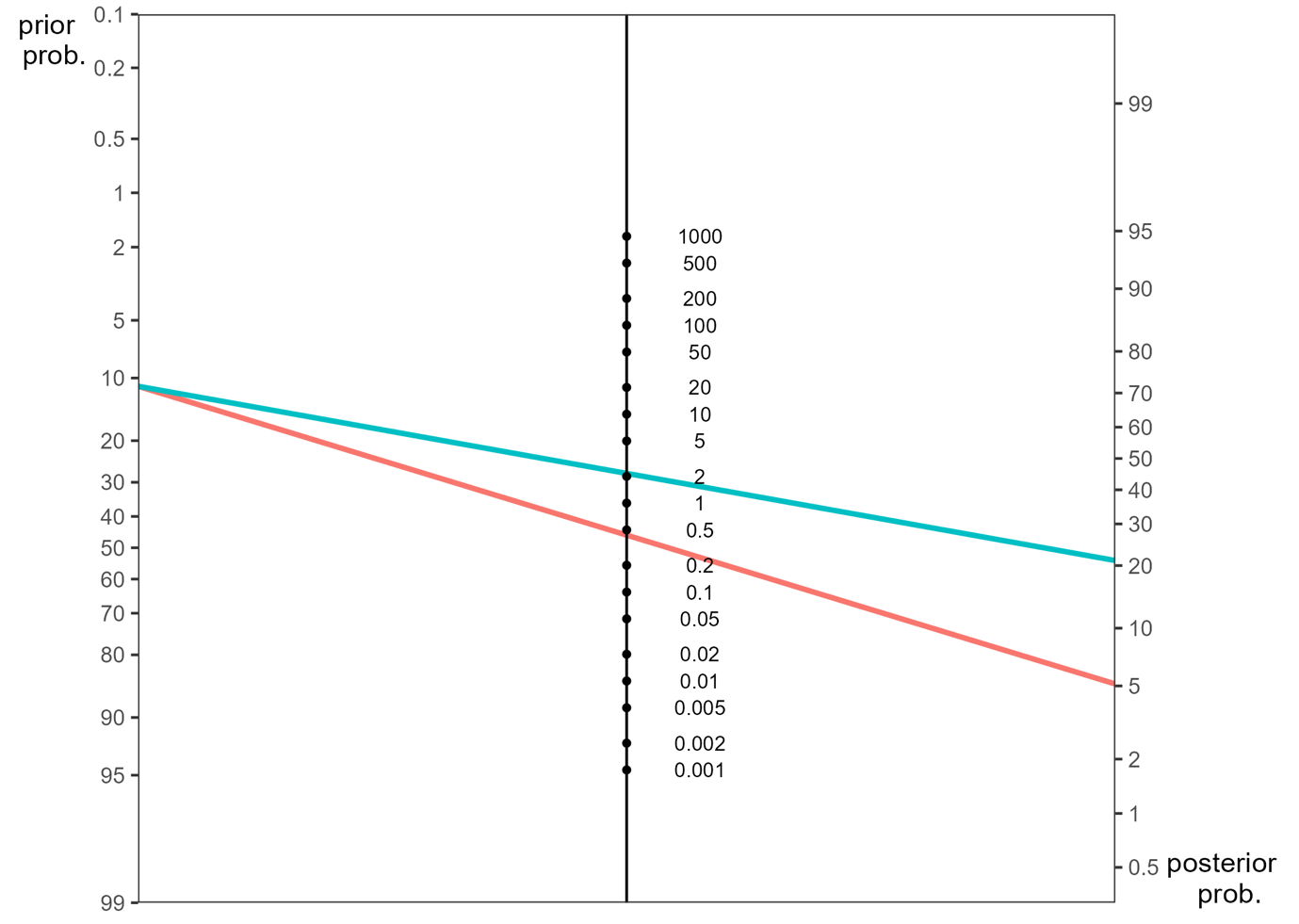


**Supplementary Figure 2:** Risk of Bias findings


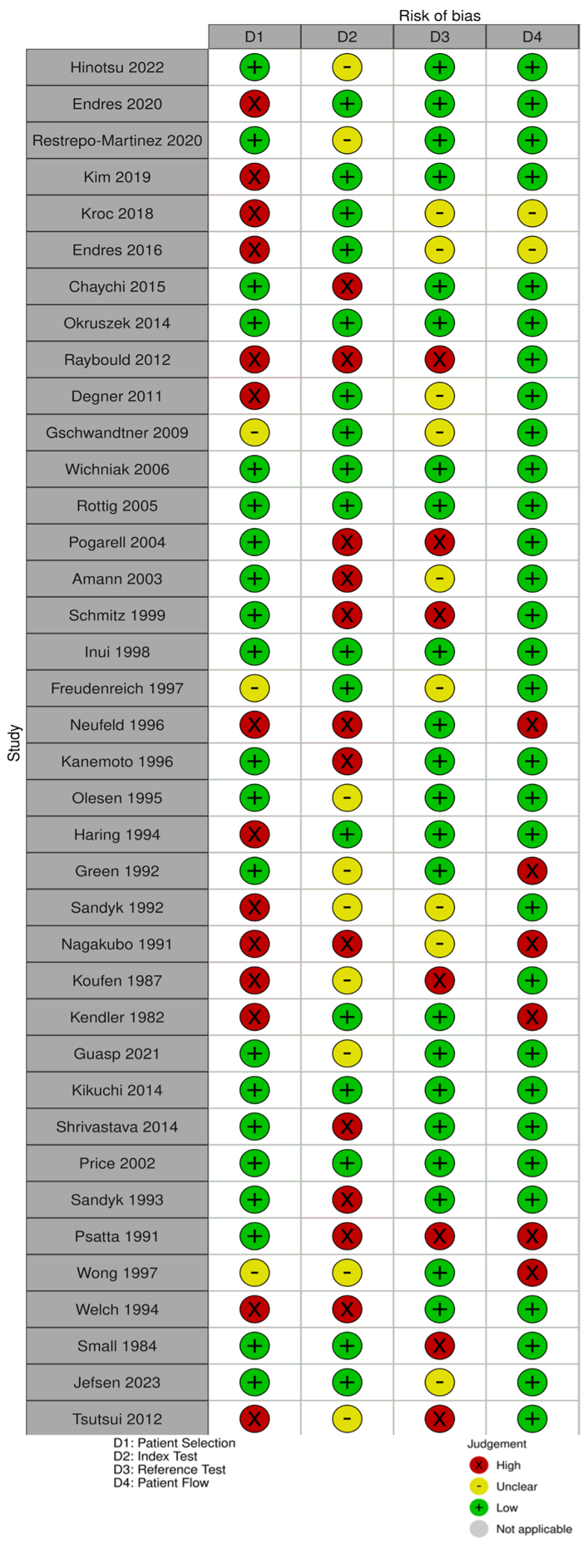

Supplement: Supplementary materials [file EMS212209-supplement-Supplementary_materials.docx]
